# The Evolutionary History of Peptidases Involved in the Processing of Organelle-Targeting Peptides

**DOI:** 10.1093/gbe/evac101

**Published:** 2022-06-27

**Authors:** Clotilde Garrido, Francis-André Wollman, Ingrid Lafontaine

**Affiliations:** UMR7141, Institut de Biologie Physico-Chimique (CNRS/Sorbonne Université), 13 Rue Pierre et Marie Curie, 75005 Paris, France; UMR7141, Institut de Biologie Physico-Chimique (CNRS/Sorbonne Université), 13 Rue Pierre et Marie Curie, 75005 Paris, France; UMR7141, Institut de Biologie Physico-Chimique (CNRS/Sorbonne Université), 13 Rue Pierre et Marie Curie, 75005 Paris, France

**Keywords:** endosymbiosis, organellogenesis, chloroplast, mitochondria, subcellular targeting, proteolysis

## Abstract

Most of the proteins present in mitochondria and chloroplasts, the organelles acquired via endosymbiotic events, are encoded in the nucleus and translated into the cytosol. Most of such nuclear-encoded proteins are specifically recognized via an N-terminal-encoded targeting peptide (TP) and imported into the organelles via a translocon machinery. Once imported, the TP is degraded by a succession of cleavage steps ensured by dedicated peptidases. Here, we retrace the evolution of the families of the mitochondrial processing peptidase (MPP), stromal processing peptidase (SPP), presequence protease (PreP), and organellar oligo-peptidase (OOP) that play a central role in TP processing and degradation across the tree of life. Their bacterial distributions are widespread but patchy, revealing unsurprisingly complex history of lateral transfers among bacteria. We provide evidence for the eukaryotic acquisition of MPP, OOP, and PreP by lateral gene transfers from bacteria at the time of the mitochondrial endosymbiosis. We show that the acquisition of SPP and of a second copy of OOP and PreP at the time of the chloroplast endosymbiosis was followed by a differential loss of one PreP paralog in photosynthetic eukaryotes. We identified some contrasting sequence conservations between bacterial and eukaryotic homologs that could reflect differences in the functional context of their peptidase activity. The close vicinity of the eukaryotic peptidases MPP and OOP to those of several bacterial pathogens, showing antimicrobial resistance, supports a scenario where such bacteria were instrumental in the establishment of the proteolytic pathway for TP degradation in organelles. The evidence for their role in the acquisition of PreP is weaker, and none is observed for SPP, although it cannot be excluded by the present study.

**Significance**

The peptidases involved in proteolysis of those peptides that allow the proper targeting of protein to mitochondria and chloroplast are of bacterial origin, but the evolutionary scenario of these acquisitions remained undetermined. Here, we compiled an exhaustive catalog of these peptidases across the tree of life and show how they were acquired once or twice, at the time of primary endosymbiosis, with the possible contributions of other bacteria than the chloroplast and the mitochondrial progenitors. These results shed new light on the origin of the degradation process of targeting peptides in organelles that may have involved bacteria resistant to antimicrobial peptides.

## Introduction

Eukaryotic organelles, mitochondria and chloroplasts, evolved from ancestral bacteria through primary endosymbiosis. Mitochondria most probably derived from α-proteobacteria (Wang and Wu 2015; Roger et al. 2017) and the chloroplast from an ancestral cyanobacterium, probably close to *Gloeomargarita litophora* (Jensen and Leister 2014; Ponce-Toledo et al. 2017). These endosymbiotic events were accompanied by massive horizontal gene transfer (HGT) of genetic material from the bacterial progenitors to the host genome that is named endosymbiotic gene transfer (EGT) (Timmis et al. 2004). Today, most of the proteins found in mitochondria and chloroplast are encoded in the nucleus, translated in the cytosol, and subsequently imported into the organelles through dedicated translocon machineries: the Translocase of the Outer/Inner mitochondrial Membrane (TOM/TIM) for mitochondria (Wiedemann and Pfanner 2017) and the Translocon at the Outer/Inner envelope Membrane of Chloroplasts (TOC/TIC) for chloroplast (Chotewutmontri et al. 2017). In most cases, this import is driven by an N-terminal targeting peptide (TP).

The degradation of TPs in organelles is ensured by zinc metallopeptidases acting on different peptides according to their lengths (Johnson et al. 2006; Kmiec and Glaser 2012). The degradation follows common steps in the two organelles (Teixeira et al. 2017; Kmiec et al. 2018). First, two endopeptidases, the mitochondrial processing peptidase (MPP) (Taylor et al. 2001) and the stromal processing peptidase (SPP), cleave the TP, respectively, in the mitochondria and the chloroplast, thus releasing the mature protein. These enzymes have critical roles in cell viability. MPP is essential for cell viability in yeast (Yaffe et al. 1985) and nematodes (Nomura et al. 2006), and mutant of the MPP catalytic subunit cause neurodegeneration in early childhood (Vögtle et al. 2018). Similarly, SPP is essential in *Arabidopsis thaliana* (Trösch and Jarvis 2011) and also has been shown to impact protein import in pea (Zhong et al. 2003). The resulting TP is further degraded by the presequence protease (PreP) that cleaves peptides ranging from 10 to 65 amino acids (Kmiec and Glaser 2012) and by the organellar oligo-peptidase (OOP) that cleaves peptides ranging from 8 to 23 residues (Kmiec et al. 2013). In photosynthetic eukaryotes, both PreP and OOP are dually targeted to mitochondria and chloroplast. To the best of our knowledge, all described PrePs are targeted to organelles, while many OOP paralogs remain in the cytosol. In mitochondria, the cleavage by MPP may be followed by an additional cleavage by Oct1, an OOP homolog (Kmiec et al. 2013), or Icp55 in order to obtain the final mature protein, more stable than the intermediate form released after the MPP cleavage (Vögtle et al. 2009, 2011; Venne et al. 2013). Finally, single amino acids are released by the action of aminopeptidases from the M1 and M17 metalloprotease families acting on peptides <8 amino acids (Kmiec et al. 2018). MPP, SPP, and PreP are members of the M16 family, whereas OOP is part of the M3 family in the MEROPS hierarchical classification (Rawlings et al. 2018). In eukaryotes, MPP is a heterodimer, composed of a catalytic subunit, MPP-β in *A. thaliana* (Mas1 in yeast and PMPCB in human) and MPP-α (Mas2 and PMPCA) (Poveda-Huertes et al. 2017). Default of TP degradation can perturb organelle integrity and biogenesis, as free peptides may have a toxic effect (Kmiec et al. 2014). Also, human PreP is responsible for the degradation of amyloid-β peptides that accumulate into mitochondria in Alzheimer’s disease patients, which saturates the capacity for peptide degradation in the mitochondrion and leads to the accumulation of immature precursor proteins (Pinho et al. 2014). In yeast, in addition to Cym1 (the yeast PreP homolog), two other M16 proteases are involved in mTP degradation: Prd1 in the intermembrane space (Kambacheld et al. 2005) and Ste23, which is also able to cleave amyloid-β peptides (Taskin et al. 2017). For a complete overview of TP processing in mitochondria or chloroplast, including additional peptidases with a narrower set of substrates, see recent reviews (van Wijk 2015; Poveda-Huertes et al. 2017; Ghifari et al. 2019).

Peptidases from the M16, M3, M1, and M17 families involved in TP cleavage and degradation are well conserved from bacteria to eukaryotes, and M16 and M3 peptidases may have been present in the Last Universal Common Ancestor (LUCA) (Rawlings et al. 2018; Rawlings and Bateman 2019). However, the MEROPS families are very large, with members carrying varied functions, because the classification is solely based on the part of the protein directly responsible for the peptidase activity (substrate binding site and the catalytic residues). Therefore, they are not adapted to accurately retrace the evolutionary history of a specific peptidase. The possible presence of an M16 and an M3 peptidase in LUCA does not imply that the functions now ensured by MPP, SPP, PreP, and OOP were present as well. Rather, previous studies all pointed for a bacterial origin of the organelle peptidases, as peptidases acting similarly are also found in bacteria, which is not the case for the protein degradation processes observed in the nucleus and the cytosol where these are mainly performed by the proteasome (Van Dyck and Langer 1999; Adam and Clarke 2002). It is of note that in photosynthetic eukaryotes, both mitochondria and chloroplasts, which arose from two distinct primary endosymbiotic events, use the same players (PreP and OOP) and players from the same peptidase family (MPP and SPP) to ensure disposal of their TPs.

We recently provided experimental evidence for an antimicrobial origin of TPs. A subclass of antimicrobial peptides (AMPs) are synthesized by the ribosome and adopt an amphipathic helical structure (HA-RAMPs) (Garrido et al. 2020), supporting the idea that the import machineries into organelles may derive from an antimicrobial resistance machinery in the bacteria involved in early endosymbiotic events (Wollman 2016; Caspari and Lafontaine 2021). In this view, the translocon machineries and the peptidases involved in the proteolytic degradation of TP may derive, respectively, from bacterial transporters and bacterial peptidases that participated in antimicrobial resistance. In agreement with this hypothesis, it has been demonstrated that MPP most probably originates from a progenitor of a rickettsiales putative peptidase (RPP) in a parasitic bacterium, and that extant bacterial RPP still have the capability to cleave mitochondrial TPs (Kitada et al. 2007). To challenge this hypothesis further, here, we focused on the peptidases involved in the first steps of the main proteolytic pathway (MPP, SPP, PreP, and OOP), leaving aside more specific peptidases, as well as the house-keeping aminopeptidases that degrade small peptides <10 amino acids, acting at the very end of the proteolytic pathway. We therefore built the exhaustive catalog of MPP, SPP, PreP, and OOP families across eukaryotes, bacteria and archaea in order to decipher accurately their evolutionary history and to estimate the possible contribution of HA-RAMP-resistant bacteria in the acquisition the proteolytic pathway degrading organelle TPs.

## Results

### An Exhaustive Catalog of Organelle Peptidase Families

We searched for homologs of MPP, SPP, PreP, and OOP in 8,340 reference proteomes from Uniprot comprising 291 archaea, 6,820 bacteria, and 1,230 eukaryotes (including 135 photosynthetic eukaryotes). The MPP, SPP, PreP, and OOP sequences of *A. thaliana* were thus used as a starting point to construct Hidden Markov Model (HMM) profiles with the retrieved homologs (supplementary table S1, Supplementary Material online). HMM profiles allowed the detection of 36,807 candidates (10,930 PreP; 2,716 SPP; 6,822 OOP; and 16,339 MPP), from which, only the 26,211 candidates that contain all the Protein Families (PFAM) motifs present in the *A. thaliana* peptidase of reference were considered its homologs (table 1).

**Table 1:** Composition of the Peptidase Families

		Archaea (291)		Bacteria (6,820)		Eukaryotes (1,230)	
	PFAM motif	*#proteins*	*#species*	*#proteins*	*#species*	*#proteins*	*#species*
MPP	M16/M16C	11	10	10,812	4,972	3,966	1,204
PreP	M16/M16C/M16C_assoc	0	0	910	854	1,037	925
SPP	M16/M16C/M16C	0	0	2,376	1,477	279	141
OOP	M3	6	6	4,694	3,624	2,120	1,071

For each peptidase, the composition in PFAM motifs is given in the first column M16: PF00675; M16C: PF05193; M16C_assoc: PF08367; M3: PF01432. The number of homologs and number of corresponding proteomes across archaea, bacteria, and eukaryotes is given in the following columns. The total number of species considered is indicated in parenthesis for each domain.

Given the paucity of those peptidases in the 291 archaeal proteomes present in the Uniprot Reference proteomes, we searched the Uniprot database to retrieve all archaeal sequences in the ca. 440 archaeal species absent from the Reference proteomes that possess either a M16 domain (429 sequences from 143 species) or a M3 domain (1,185 sequences from 623 species) and selected homologs of MPP, PreP, and OOP following the procedure described above. We also searched homologs in the nonredundant (nr) database at NCBI (see Methods). The outcome of this search is provided in supplementary data S1, Supplementary Material online. In total, we found peptidase homologs in <7% of the 1,937 different archaea species with sequences referenced in the three databases. Among the 44 referenced Asgard species, which are considered the most closely related to eukaryotes, only one has an MPP homolog: *Candidatus Thorarchaeota archaeon* (strain OWC), and another one has an OOP homolog: *Heimdallarchaeota archaeon* (strain LC_2). PreP and SPP are absent from Asgard species.

On the proximity graph of the four peptidase families based on the BLAST pairwise *E*-value (supplementary fig. S1, Supplementary Material online), MPP, PreP, and SPP group together within the M16 peptidases families, well apart from OOP, belonging to the M3 family. Each family of M16 peptidase localizes differently from the two others, with an overlap between MPP and SPP.

### The M16 Family

**Fig. 1.**
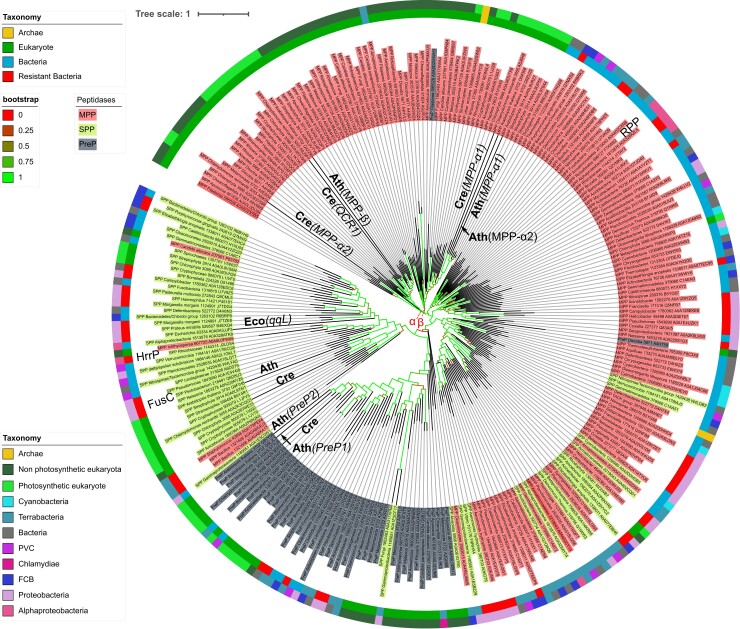
Tree of a subset of M16 peptidases with 160 MPP, 60 SPP, and 44 PreP sequences used (see Methods). Branches are colored according to their bootstrap value (upper left box legend). The eukaryotic MPP subtree is divided into two subtrees, α and β, indicated with red dotted arrows. The inner circle gives the classification (upper left box legend) of the sequences among the three domains of life, with a distinction for bacteria with an experimentally documented resistance to AMP (supplementary data S1, Supplementary Material online). The outer circle gives a further classification (lower left box legend) distinguishing the taxonomic groups of interest in this study. Bacterial peptidases whose functions are discussed in the text are indicated, as well as homologs from model organisms present in the subset, with their names in parenthesis for bacteria and if paralogs exist for a given peptidase in eukaryotes. Cre: *Chlamydomonas reinhardtii,* Ath: *Arabidopsis thaliana,* Eco: *Escherichia coli*. Note that in this tree, the MPP-α2 of *C. reinhardtii* groups in the β subtree, while it groups correctly in the MPP-α subtree of figure 3.

To better analyze the relationships among the peptidases of the M16 family, we built a phylogenetic tree of the 264 M16 homologs from the sample set (see Methods), based on the multiple alignment of the two first conserved domains (M16 and M16C) between MPP, PreP, and SPP (fig. 1). Each peptidase groups in three different subtrees in which both eukaryotic and bacterial homologs are found. The eukaryotic MPP subtree is divided into two subtrees, in line with an early duplication at the basis of the eukaryotic lineage, leading to the two subunits (a) and (b) of the MPP heterodimer. Only 28 homologs are misclassified on the tree: 2 PrePs and 16 SPPs localize within the MPP subtree; 1 MPP and 3 SPPs are found within the PreP subtree and 5 MPPs are found within the SPP subtree. Given that the tree is based on the first two conserved domains, and not the entire protein sequence, this could be due to sequences experiencing contrasting evolutionary rates. The rickettsiales putative peptidase (RPP), putative MPP ancestral progenitor, groups within the MPP subtree, confirming previous reports (Kitada et al. 2007). The bacterial metalloproteases FusC and HrrP that play a role in antimicrobial resistance mechanisms in Gram-negative bacteria group within the SPP subtree.

### MPP, PreP, SPP, and OOP have a Bacterial Origin

**Fig. 2.**
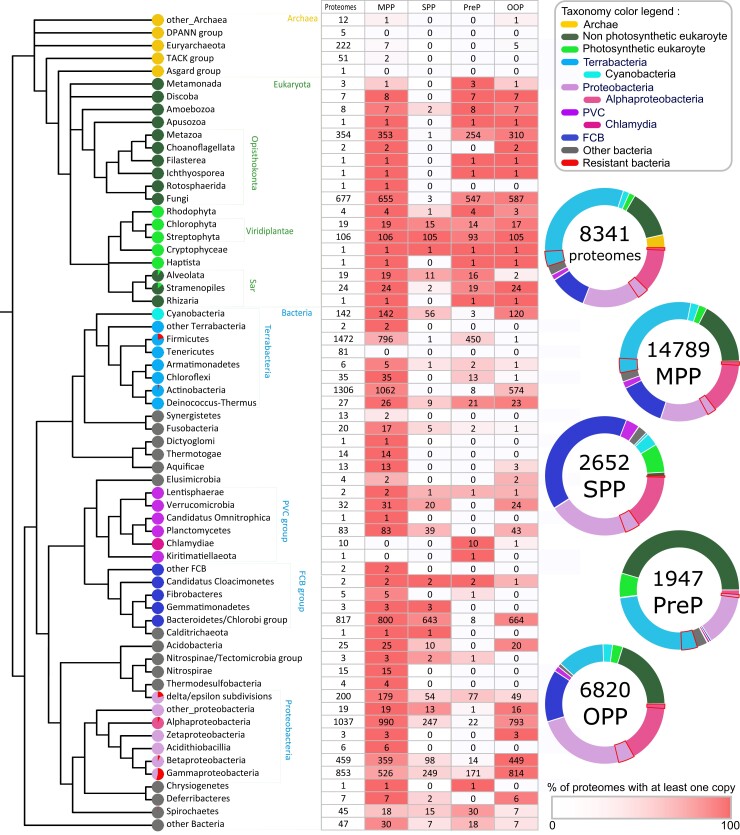
Taxonomic distribution of the four peptidases among the tree of life. The cladogram represents the phylogenetic relationships between the different taxonomic groups (see Methods for the reference trees used). Circles next to leaves are colored according to the main groups of interest, with the proportion of AMP-resistant bacteria indicated in red. Minor bacterial phyla are colored in grey. Eukaryotes are divided into photosynthetic and nonphotosynthetic groups, according to the presence or the absence of a chloroplast. In the table, the first column indicates the number of proteomes included in each group. The number of proteomes where at least one homolog is found for MPP, PreP, SPP, and OOP are given in columns 2–5, respectively. The color gradient is proportional to the number of proteomes in the group with at least one homolog of the peptidase. On the right, the upper circle indicates the distribution of proteomes in the different groups; the circles below indicate the distribution of each peptidase among the different groups. PVC: superphylum composed of Planctomycetales, Verrucomicrobia, and Chlamydiales. FCB: superphylum composed of Fibrobacteres, Chlorobi, and Bacteroidetes. The color legend for the taxonomy is given at the upper right of the figure. For SPP, the homologs retrieved in Metazoa (1) and in Fungi (3) differ from those found in photosynthetic eukaryotes by an additional M16M PFAM domain that may impact their function (see text and supplementary data S2, Supplementary Material online).

Figure 2 describes the repartition of the homologs across the tree of life. MPP, PreP, and OOP peptidases are found, respectively, in 98%, 87%, and 75% of the eukaryotic proteomes, with 69% of the studied eukaryotic species possessing the three peptidases. The Rotosphaerida species *Fonticula alba* bears only the MPP and the two Choanoflagellata species lack PreP. Their frequencies are lower in bacterial lineages 72%, 53%, and 12% for MPP, OOP, and PreP, respectively, where they are heterogeneously distributed. There are very few homologs in Archaea: MPP and OOP are present only in 16 out of 291 archaeal proteomes and no PreP was found in any of these proteomes. They are also scarce in viruses, with only 13 MPPs, 2 OOPs, and no PreP among the 9,424 viral proteomes.

SPP is the least abundant peptidase, absent from archaea and viruses, present in only 21% of the bacterial proteomes and found only in photosynthetic eukaryotes (11% of the studied eukaryotic proteomes, including two of the three photosynthetic Stramenopiles present in our proteome database) and in nonphotosynthetic eukaryotes containing a plastid like some Apicomplexa, Amoebozoa, and Alveolata species.

The taxonomic distribution of the four eukaryotic peptidases suggests that they most likely have a bacterial origin and that they were acquired early in eukaryote evolution. Only 23/1,230 (2%) eukaryotes are devoid of those four peptidases, including the 19 Microsporidia having lost their mitochondria, the parasitic nematode *Brugia timori*, the neuropathogenic flatworm *Trichobilharzia regenti*, as well as two Basidiomycota Fungi that are plant pathogens: the brown-rot fungus *Postia placenta* and *Moniliophthora perniciosa*. There are 9% (607 of 6819) of bacterial proteomes free of MPP, PreP, SPP, and OPP, among which there are four groups in which all species lack the peptidases: the 77 Mollicutes pathogens (55 Mycoplasmatales and 22 Entomoplasmatales), the 22 Enterococcus pathogenic species, and 48 Bifidobacteriales. The other 460 species are scattered among their respective groups.

### The Evolutionary History of Peptidase Families

To get insights into the evolutionary history of each peptidase family from bacteria to eukaryotes, we built its phylogenetic tree and looked for the distribution of the bacterial sequences that are close to eukaryotic homologs, which are the potential donors through gene transfers.

#### MPP and SPP, the Cleavage Peptidases, were Inherited from a Single HGT

**Fig. 3.**
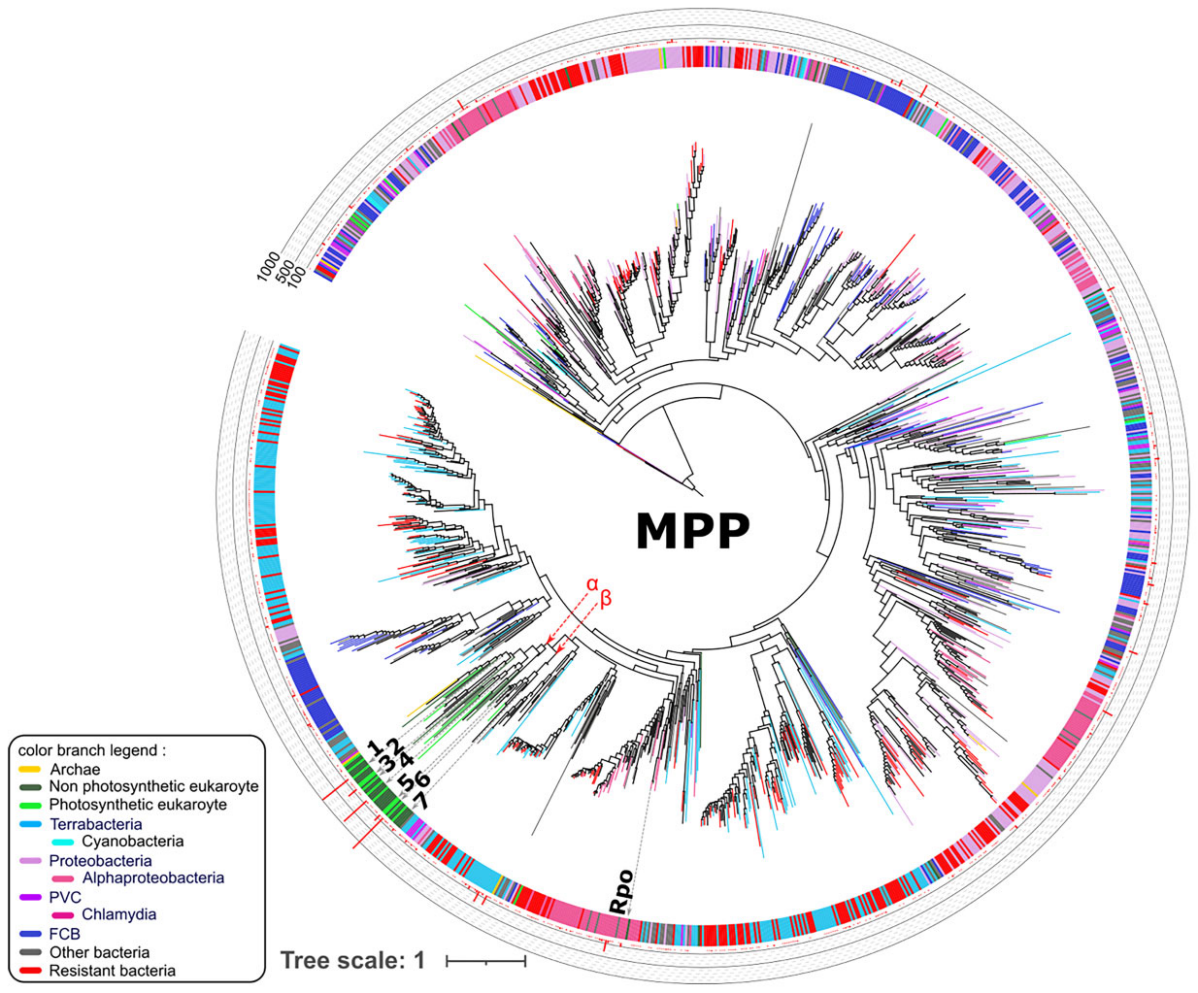
The MPP gene tree reveals a single bacterial origin of the eukaryotic MPPs. Because the entire MPP family contain 14,789 sequences, the nodes whose leaves belong to the same NCBI taxonomical group are collapsed, thus only 1,726 leaves are visible on the tree. Branches are colored according to the classification given in the left lower part of the figure. Branch supports are given in the original tree in our github repository (see Methods). The inner circle recapitulates the taxonomy. Number of sequences collapsed at each node is given on the external circle. Numbers under the inner circles indicate collapsed branches containing homologs from model organisms (the genetic loci are given). (1) *Caenorhabditis elegans* (mppa-1), *Homo sapiens* (PMPCA), *Drosophila melanogaster* (CG8728), *Saccharomyces cerevisiae* (MAS2); (2) *Arabidopsis thaliana* (MPPA1, MPPA2), *Chlamydomonas reinhardtii* (MPPA1); (3) *C. reinhardtii* (MPPA2); (4) *C. elegans* (ucr-2.2, ucr-2.1), *H. sapiens* (UQCR-C2), *D. melanogaster* (UQCR-C2); (5) *A. thaliana* (MPP-β), *C. reinhardtii* (QCR1); (6) *C. elegans* (mppb-1, ucr-1), *H. sapiens* (PMPCB, UQCR-C1), *S. cerevisiae* (MAS1), *D. melanogaster* (UQCR-C1); (7) *S. cerevisiae* (COR1). Rpo is the homolog found in *Rickettsia prowazekii*. The red dotted arrows indicate the two eukaryotic MPP subtrees: (α) containing the collapsed branches 1–4 and (β) containing the collapsed branches 5–7.

On the MPP tree, all the eukaryotic MPP sequences form a monophyletic group among other bacteria (fig. 3), which suggests that the ancestral eukaryotic MPP has been acquired by a single HGT. Note that because there are more than 14,000 MPP homologs, the tree is collapsed. The eukaryote MPP subtree forms a monophyletic group with some homologs from α-proteobacteria (see supplementary fig. S2, Supplementary Material online for a tree with a sampling of bacterial homologs), in agreement with an EGT from the mitochondrial progenitor during or following the early stages of mitochondrial endosymbiosis. In some bacterial homologs, the M16–M16C region is repeated. Given their heterogeneous distribution on the tree presented in supplementary fig. S3*A*, Supplementary Material online, it is unlikely that it represents an ancestral form of MPP. 132 additional PFAM domains were identified in our retrieved candidates. These additional domains are heteregeneously distributed and shared by <8 species each, with two exceptions found in the leotiomyceta clade in the Ascomycota phylum (supplementary data S2, Supplementary Material online). The addition of these domains could suggest two independent subfunctionalizations, one in Eurotiomycetes and one in Leotyomycetes.

**Fig. 4.**
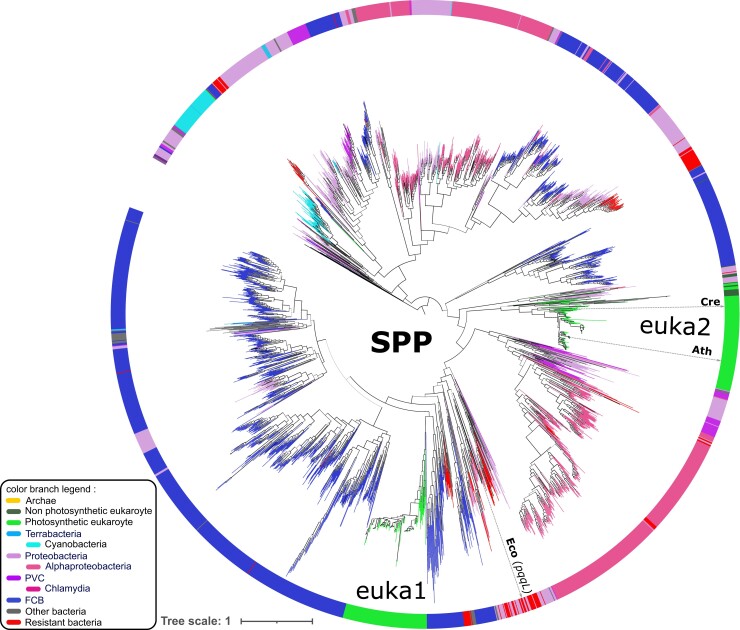
Phylogenetic tree of the 2,652 SPP homologs. Five additional sequences are used as outgroup (see supplementary data S1, Supplementary Material online). Branches are colored according to the classification given in the left lower part of the figure. The inner circle recapitulates the taxonomy. Branches with support higher than 0.8 are depicted in bold. Homologs from model organisms are indicated next to the inner circle, with the name of the *Escherichia coli* homolog in parenthesis. Cre: *Chlamydomonas reinhardtii,* Ath: *Arabidopsis thaliana*, Eco: *E. coli*. See supplementary figure 3*A*, Supplementary Material online for a collapsed version of this tree.

On the SPP tree, homologs from photosynthetic eukaryotes are divided into two subtrees (fig. 4). A first one as a sister group of sequences from the superphylum composed of Fibrobacteres, Chlorobi, and Bacteroidetes (FCB) in the lower part of the tree (euka1), and a second one at the basis of the larger group containing the euka1 subtree (euka2). The majority (93/141) of eukaryotic species have a paralog in both subtrees. This topology can be explained by an acquisition from a FCB bacteria by HGT at the basis of the photosynthetic eukaryotic lineage, followed by a duplication event and the divergent evolution of one of the paralogous copies. The conservation of the domain architecture of the SPP homologs from the lower subtree containing the euka1 and euka2 subtrees (supplementary fig. S3*B*, Supplementary Material online) supports this hypothesis. Note that homologs from euka2 appear to bear an additional N-terminal part, compared to all other homologs from the lower subtree that could explain their basal position. Noticeably, there are very few SPP homologs in cyanobacteria (64 from the 142 cyanobacterial proteomes studied), most closely related to chloroplast, and they form a monophyletic group distant from the euka1 and euka2 subtrees. 33 species (28 bacteria and 5 eukaryotes) and 8 others (7 bacteria and 1 eukaryote) have an additional M16M and M16C_assoc PFAM domain in their identified SPP homologs, respectively. The other 26 additional PFAM domains in SPP candidates are found in <4 species each (supplementary data S2, Supplementary Material online).

#### OOP and PreP Degrade the TP after their Cleavage has been Acquired Twice in Close Relationship with the Two Primary Endosymbiosis

**Fig. 5.**
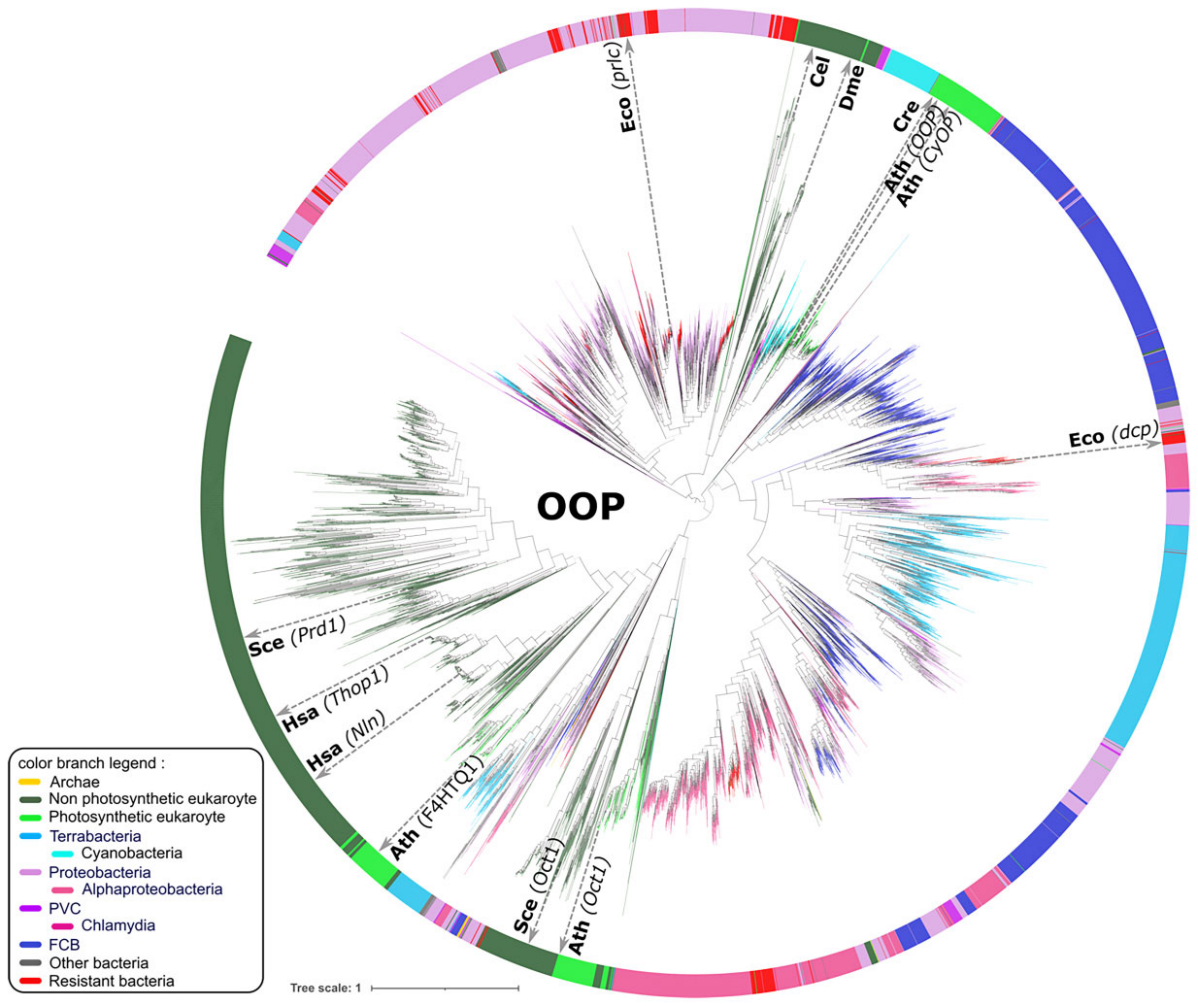
Phylogenetic tree of the 6,820 OOP homologs, rooted at midpoint. Homologs from model organisms are indicated next to the inner circle, with their name in parenthesis for bacterial homologs if paralogs exist in eukaryotes. Cre: *Chlamydomonas reinhardtii,* Ath: *Arabidopsis thaliana*, Eco: *Escherichia coli*. Hsa: *Homo sapiens*, Sce: *Saccharomyces cerevisiae*, Dme: *Drosophila melanogaster*. See supplementary figure 3*B*, Supplementary Material online for a collapsed version of this tree.

On the complete OOP tree (fig. 5), a large group of 1,393 eukaryotic sequences, 110 of which are from photosynthetic eukaryotes, form a sister group in the lower left subtree with mainly Proteobacteria and Terrabacteria, suggesting an acquisition from these bacteria at an early step of eukaryogenesis. Another 171 sequences from photosynthetic eukaryotes, 120 cyanobacteria, 15 PVC, 5 Proteobacteria, and 1 Chlamydiales, a potential partner of the chloroplast endosymbiosis (Ball et al. 2016) form a monophyletic group in the upper left part of the tree (fig. 5), suggesting a second acquisition of a bacterial OOP during chloroplast endosymbiosis. The majority of photosynthetic eukaryotes (85 over 123) has a paralog in both eukaryotic subtrees, thus they conserved the two acquired OOP copies. As the OOP family contains many paralogs (172 in eukaryotes on average) some of which being cytosolic (three in *A. thaliana*), we built a tree with eukaryotic OOP that are predicted to be addressed to either the chloroplast or the mitochondria and their closest bacterial homologs (one per species). The taxonomic distribution of the peptidases remains unchanged and so are the evolutionary conclusions (supplementary fig. S5, Supplementary Material online). 135 additional PFAM domains were found in OOP candidates, found in <10 species each (supplementary data S2, Supplementary Material online).

**Fig. 6.**
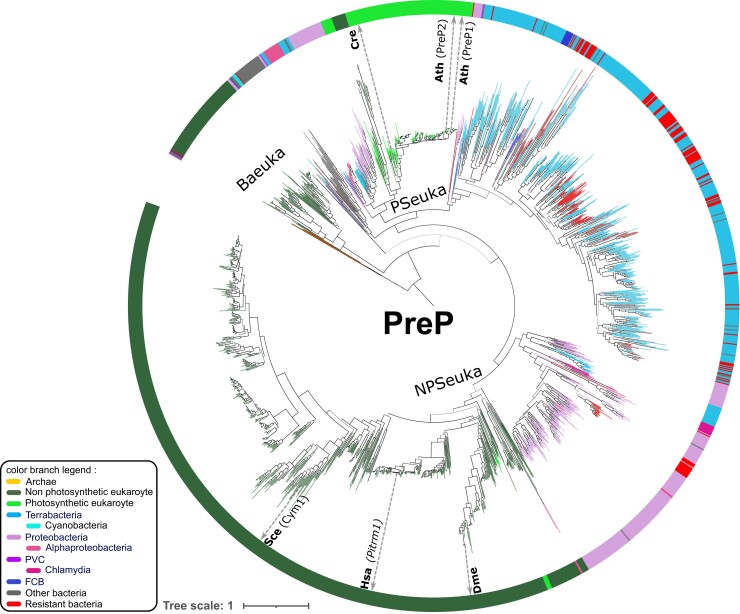
Phylogenetic tree of the 1,947 PreP homologs. Homologs from model organisms are indicated next to the inner circle, with their name in parenthesis unless there are only systematic names. See supplementary figure 3*C*, Supplementary Material online for a collapsed version of this tree.

The eukaryotic PreP homologs are distributed among three subtrees (fig. 6). The vast majority of homologs from nonphotosynthetic eukaryotes (770) form a monophyletic group with a sister bacterial clade mainly composed of Proteobacteria (NPSeuka subtree, fig. 6) and all but 5 (148) PreP from photosynthetic eukaryotes form a monophyletic group with a sister bacterial clade composed of homologs form Proteobacteria and Terrabacteria (PSeuka subtree, fig. 6). Another 97 Opisthokonta sequences (from 91 distinct species) form a long-branched group at the basis of the tree (Baseuka subtree, fig. 6), suggesting they are highly diverged homologs that could be related to either the NPSeuka or the Pseuka subtree. Ignoring the Baseuka subtree, the tree topology suggests two independent acquisitions of PreP in eukaryotes: one associated with the mitochondrial endosymbiosis (within the NPSeuka subtree) and a posterior one that could be associated to the chloroplast endosymbiosis (within the Pseuka subtree). Among bacteria, PreP is present most exclusively in Terrabacteria (493 homologs) and in Proteobacteria (263) (fig. 2 and supplementary data S1, Supplementary Material online), containing the cyanobacteria and α-proteobacteria, respectively, the extant bacteria most closely related to chloroplast and mitochondria. The nearly complete absence of homologs from photosynthetic eukaryotes in the NPSeuka subtree suggests that the PreP copy acquired at the time of the mitochondrial endosymbiosis has been lost after the acquisition of the chloroplast, except in five microalgae. The timing of the second PreP acquisition relatively to the chloroplast endosymbiosis is difficult to determine with the present data when considering the Baseuka subtree: half of these Opisthokonta homologs correspond to paralogs from the NPSeuka subtree, supporting an independent duplication before the Opisthokonta divergence followed by rapid evolution, and a second PreP acquisition during or after the chloroplast endosymbiosis. But the remaining half sequences in the Baseuka subtree have no paralog in the NPSeuka subtree and could also correspond to highly diverged homologs from the Pseuka subtree. This would imply an alternative, but less parsimonious scenario, in which the two acquisitions of PreP occurred before the chloroplast endosymbiosis so that ancestral eukaryotes had two PrePs and one copy has been differentially lost depending on the presence of a chloroplast in the majority of the lineages. In support of this alternative scenario, the domain composition of PreP homologs in the Baseuka subtree is identical to the one in the Pseuka subtree (supplementary fig. S3*C*, Supplementary Material online). 27 additional PFAM domains were found in PreP candidates, found in <7 species each (supplementary data S2, Supplementary Material online).

#### Molecular Evolution of the Peptidase Motifs from Bacteria to Eukaryotes

**Fig. 7.**
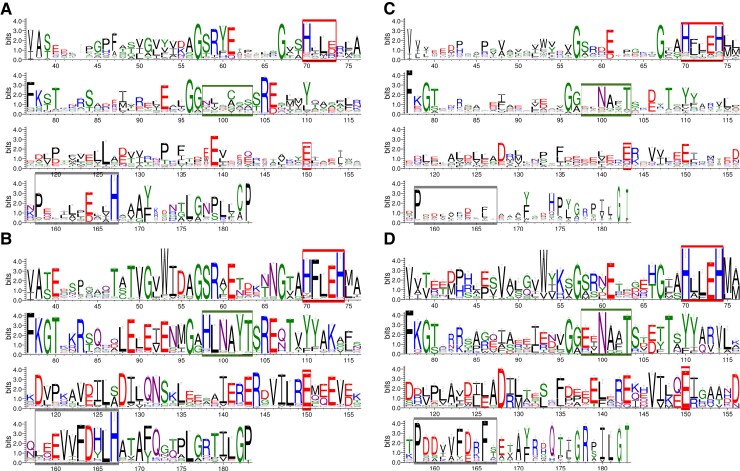
Sequence logos of the M16 Motif in MPP homologs. (*A*) MPP-α eukaryotic homologs (2,306). (*B*) MPP-β eukaryotic homologs (1,633). (*C*) All MMP bacterial homologs (10,812). (*D*) The closest to eukaryotes 200 bacterial homologs according the similarity distance (top200 bacteria). The positions are those of the M16 motif in the catalytic subunit (MPP-β) of *Saccharomyces cerevisiae,* Mas1. The overall height of the stack at each position reflects conservation, the height of residues reflects their relative frequency and the width of the stack is inversely proportional to the number of gaps at that position. Residues are colored according to their chemical properties, green: polar, purple: neutral, basic: blue, acidic: red, and hydrophobic: black. The position of the HXXEHX_76_E motif of the catalytic site in MPP- β is indicated by red boxes in each panel. The green box indicates the position of the beta strand involved in the binding cavity of the MPP heterodimer (Taylor et al. 2001): strand 1 at the catalytic site in MPP-β and strand 4 in MPP-α, as shown in figure 8. The grey box indicates positions discussed in the text.

To get further insights into the evolution of the function of these peptidases, we compared the conserved motifs containing their catalytic sites. The logo representations of the multiple alignments of the M16 motif in the MPP family are presented in figure 7. Eukaryotic homologs in the MPP tree were annotated as MPP-α or MPP-β according to their localization in either the subtree α or the subtree β (fig. 3). The 27 MPP homologs grouping outside these two subtrees where not considered.

**Fig. 8.**
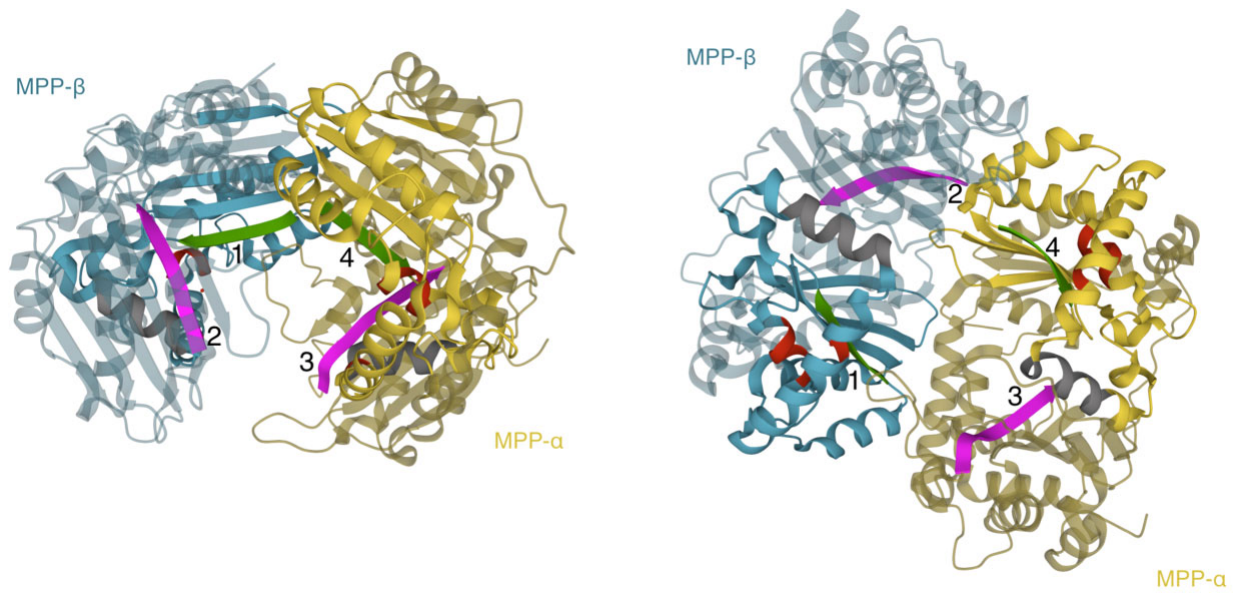
The heterodimeric yeast MPP. Crystal structure of the yeast MPP (Taylor et al. 2001, PDB:1hr6) in two different orientations. MPP-β (Mas1) is drawn in blue and MPP-α (Mas2) in yellow. In each subunit, the M16 region is in brighter color. The position of the HXXEHX_76_E motif found in MPP-β is depicted in red in both subunits. The region in the grey box of figure 7 is depicted in grey. Beta strands delimiting the binding cavity are numbered 14. Green: strands 1 and 4 in the M16 motif, magenta: strands 2 and 3 in the M16C motif. This figure was created with the RCSB PDB 3D viewer.

The logos for MPP-β (fig. 7*B*) and for MPP in the 200 bacteria that are the most closely related to eukaryotic homologs, hereafter referred to as top200-bacteria (fig. 7*D*) show a much higher overall conservation (indicated by the height of the stacks in the logo) than those of MPP-α (fig. 7*A*) and MPPs in the bulk of bacteria (fig. 7*C*). Further comparison of the logos on figure 7*B* and *D* shows however that the most frequent amino acid at a given position is not conserved in most cases. A noticeable exception is the zinc-binding motif HXXEHX_76_E (red boxes in fig. 7) required for the activity of the catalytic subunit MPP-β (fig. 7*B*) which is well conserved in the top200-bacteria and in the bulk of bacterial homologs (fig. 7*C* and *D*), suggesting that it was already present in the bacterial donors of MPP. Note that HXXEH has been lost in MPP-α which is consistent with it being an inactive form in the MPP heterodimer found in eukaryotes (fig. 8).

Contrary to the eukaryotic MPPs, bacterial ones do not function in a heterodimeric context. A possible signature of this functional shift from bacteria to eukaryotes can be found in the HXXEH motif that reads HFLEH in MPP-β but HLLEH in the MPP from the top200-bacteria (fig. 7*B* and *C*). Also, the positions 98–103 (green boxes, fig. 7) read HLNAYT in MPP-β but EXNAXT in the top200-bacteria and XXNAXT in the bulk of bacteria. In MPP-β, this region corresponds to the beta strand located at the active site (strand 1, fig. 8) within the binding region of the mitochondrial TP, delimited by the edges of four β sheets (Taylor et al. 2001). In the inactive MPP-α, there is no conservation at these positions, which correspond to strand 4 of the substrate binding region (fig. 8). Several other changes in the logos between MPP-β and bacterial MPPs are also likely to be related to the functional shift from bacteria to eukaryotes, like positions 158–167 (grey box, fig. 7) that are not conserved in the bulk of bacterial MPPs except for the proline at position 158, read PDDVVFDRFX in the top200-bacteria but becomes EEVVFDHLH in MPP-β. Logos of the multiple alignments for the other peptidases are given in supplementary figures S6, S7, S8, Supplementary Material online. Similar observations suggesting a possible functional shift from bacteria to eukaryotes can be made for OOP and SPP, but not for PreP.

### Some Peptidases from AMP-Resistant Bacteria are Close to their Eukaryotic Homologs

Next, we estimated the extent at which AMP-resistant bacteria could have been involved in the acquisition of the peptidases from the proteolytic degradation pathway of organellar TP. It is worth mentioning here that our definition of AMP-resistant bacteria is restricted to those bacteria for which there are publications that document an AMP-resistance experimentally. A number of bacteria has not been studied yet in that respect.

**Fig. 9.**
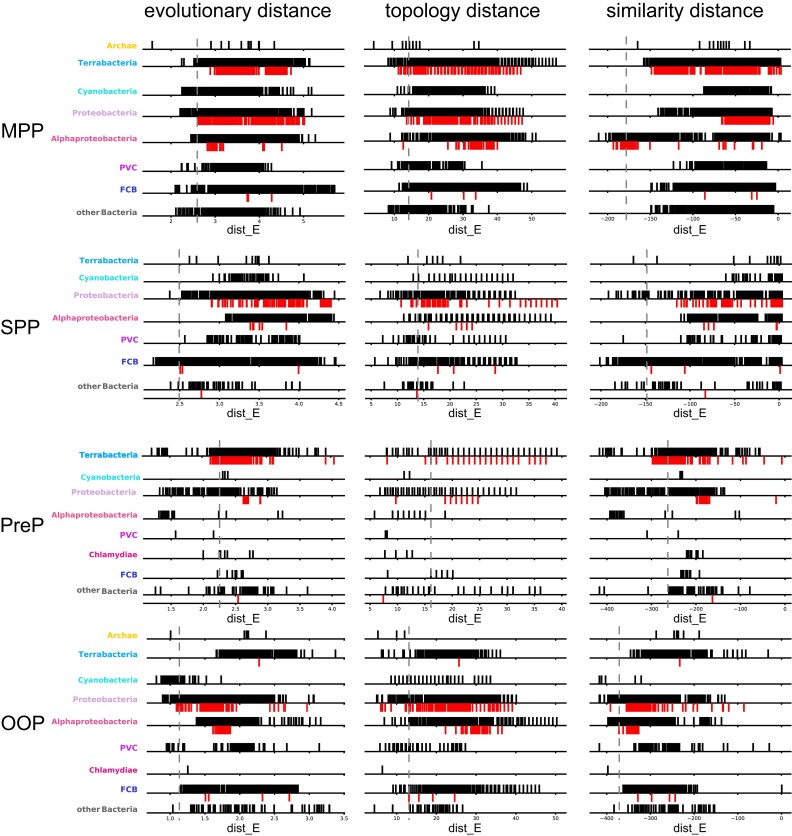
Distribution of the distance of each bacterial peptidase to its closest eukaryote homolog (dist_E, see Methods). The evolutionary topology and similarity distances are given in the first, second, and third panels, respectively. As the conservation varies among bacterial groups for the four different peptidases, the number of distributions also varies. On the left of the dashed line, are the 200 bacterial homologs most closely related to their eukaryotic counterparts.

For each peptidase family, we determined the distance of each bacterium to its closest eukaryotic homolog according to three different metrics: 1) the evolutionary distance between each pair of species, that is, the sum of branch length separating two leaves of the complete gene family tree, 2) the topological distance between each pair of species, corresponding to the number of internal nodes separating a pair of species on the complete gene family tree, and 3) the log(*E*-value) of the best Blast hit between two sequences (see Methods). We took these three metrics into account to avoid erroneous conclusions due to phylogenetic reconstruction uncertainties, considering they will be somehow compensated. Figure 9 presents the evolutionary distance of all bacterial sequences to their closest eukaryote counterparts. We defined as the top200 set, the 200 bacteria that are most proximal to their eukaryotic homologs in each group, for a given metric, and pan-top200 corresponds to the union of the top200 sets of three metrics (supplementary table S2, Supplementary Material online).

For the MPP and OOP families, homologs from resistant-bacteria of a given group are distributed closer to their eukaryotic counterpart than the other bacteria of that group. This is the case for the MPP homologs of α-proteobacteria according to both the evolutionary and the pairwise distances (fig. 9, upper panel). This is also the case for the OOP homologs from resistant proteobacteria according to all three distances (fig. 9, lower panel). On the contrary, PreP from resistant-bacteria is more distantly distributed to eukaryotes than the other bacterial PreP (fig. 9, second panel) and there is no bias in the distribution of the SPP homologs in resistant-bacteria (fig. 9, third panel). Still, we note the presence of several resistant-bacteria close enough to their eukaryotic counterparts as will be discussed below.

For the MPP family, 22% (114/521) of homologs of the pan-top200 belongs to resistant-bacteria among which 46 *Pseudomonas* and 35 *Rhizobium* (α-proteobacteria). For the OOP family, 10% (40/402) of homologs of the pan-top200 belongs to resistant-bacteria, mainly γ-proteobacteria, with 30 Enterobacteria and 8 Pseudomonadales. Also, a significant part (85%) of the homologs from resistant proteobacteria groups with the eukaryote subtree at the upper left (fig. 6). For the PreP family, even if the distribution of homologs from AMP-resistant bacteria is unbiased, 57 are in the pan-top200 (16.7% of them), among which 44 from Clostridium species. For the SPP, there are only nine homologs from resistant-bacteria (8 γ-proteobacteria and *Borreliella burgdorferi*) in the pan-top200 (2% of them) (fig. 9, third panel).

At the genus level, *Pseudomonas* species have homologs in the pan-top200 of MPP, OOP, and SPP, and Acinetobacter have close homologs in the pan-top200 of SPP, PreP, and OOP. *Borreliella* species have homologs in the pan-top200 of SPP and PreP; *Escherichia* and *Acinetobacter* species have homologs in the pan-top200 of OOP and SPP. However, only few bacterial species have peptidases homologs in the top-200 of more than one family. The most notable case is four *Pseudomonas* species (*P. agarici*, *P. cremoricolorata*, *P. fluorescens F113* and *P. sp. 5*) with a homolog in the OOP pan-top200 and a homolog in the MPP pan-top200.

Regardless of their distance to eukaryotic homologs, co-occurences of pairs of the four peptidases in AMP-resistant bacteria are significantly less frequent than observed in the whole bacterial clade. However, the most frequent co-occurrence of peptidases among the 658 AMP-resistant bacteria is observed for OOP and MPP, in 167 species (25%), notably in all studied *Pseudomonas* and FCB species, and in the majority of the α and γ-proteobacteria. This co-occurence of OOP and MPP is significantly higher in resistant α−proteobacteria than in non-resistant α−proteobacteria. Similarly, the co-occurence of MPP and PreP is higher in resistant Terrabacteria than in non-resistant Terrabacteria. The co-occurence of OOP and SPP is observed in 58 AMP-resistant bacterial species (8%), including 28 Vibrio. The co-occurence of PreP and OOP is observed only in the 15 Acinetobacter species (supplementary fig. S9, Supplementary Material online). Note that only 98 out of the 658 AMP-resistant bacteria studied (15%) are devoid of homolog for the four studied peptidases.

## Discussion

**Fig. 10.**
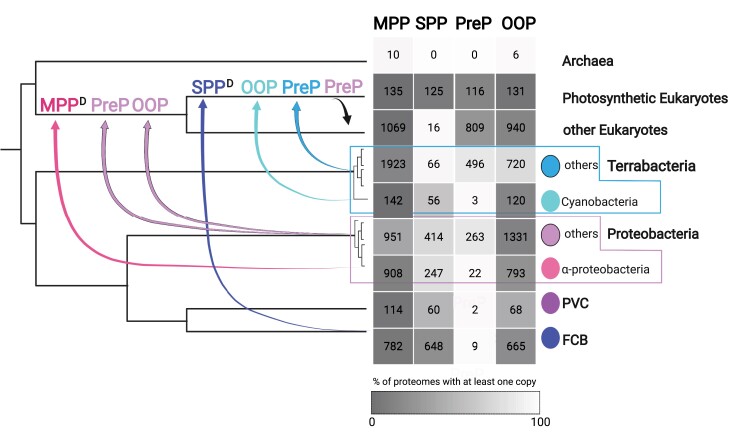
Scenario of gains and losses of the four peptidases across the tree of life. Up arrows represent horizontal transfer. Peptidase names and up arrows are colored according to the bacterial donor group. Down arrows represent loss. Superscript “D” indicates a basal duplication in the group. In the table, the number of proteomes containing at least one homolog of each peptidase is indicated. The heatmap indicates the percentage of the proteomes with at least one homolog within each group. This figure has been created with Biorender.com.

We established a complete version of the homologous families of the peptidases involved in the proteolytic pathway of TPs in mitochondria and chloroplast, recapitulated in figure 10. Indeed, deep phylogenies are based on very ancient signal and incorrectly account for the very different modes of genetic transmission between bacteria and eukaryotes, and are therefore hardly devoid of errors. In an attempt to avoid, as much as we could, erroneous conclusions due to phylogenetic artifacts, we used a simple strategy-mixing metrics based on evolutionary models and a simple similarity distance. If our phylogenies are error-free, our results confirm that MPP, PreP, SPP, and OPP were inherited from bacterial ancestors, but not necessarily the organelle progenitors. Indeed, we found no SPP and PreP homologs in Archaea and less than a dozen of archaeal MPP and OOP homologs. The scarce distribution on the archaeal homolog on the MPP and OOP gene trees provide no evidence for their presence in LUCA, but rather for an acquisition from bacteria. Such HGT from Bacteria to Archaea are estimated to be 5-fold more frequent than HGT from Archaea to Bacteria, and have been largely documented, see, for example, Nelson-Sathi et al. (2015).

In the presentation of our results, we assumed that HGT occurred shortly after or during the endosymbiosis, directly from ancestral bacteria close to the extant ones, to the ancestral eukaryotes. This implies that bacterial partners other than the endosymbiont ancestors would have been involved in such events, as proposed earlier (Gray 2015; Ball et al. 2016; López-García and Moreira 2020). Another and non-mutually exclusive possibility is that the ancestors of mitochondria and chloroplast have transferred to the host cell part of their ancestral pan-genome, that is, the entire gene set of their species, which was different from the present pan-genome. Thus, the present phylogenetic proximities observed with bacteria other than cyanobacteria and proteobacteria could also be caused by posterior HGT events among prokaryotes and may reflect that they were part of the ancestral pan-genome of mitochondria and chloroplast precursors (Ku et al. 2015). Whatever the actual source of these transfers, genetic properties that are absent from the present repertoire of extant bacteria close to the endosymbiont ancestors are likely to have played an important role for the establishment and the maintenance of the endosymbionts, notably from pathogenic partners that helped to escape host defense mechanism. Also, we found several molecular signatures specific of either bacterial or eukaryotic homologs, which could play a role in the functional shift in their activity in eukaryotes, except for PreP. Further biochemical investigations should be undertaken to assess the significance of these observations.

Our results suggest that some AMP-resistant bacteria could have contributed to the setup of the TP proteolytic pathway. MPP was most probably inherited by EGT from an α-proteobacteria-like mitochondrion ancestor, most probably pathogenic. This is in line with previous findings that the rickettsiales putative peptidase (RPP) in the obligate intracellular *Rickettsialles* is able to cleave mTP from a couple of yeast, human, and mouse preproteins. Most notably, RPP can induce the peptidase activity of the yeast MPP-β subunit for the processing of proteins, that MPP-β normally does not perform (Kitada et al. 2007). It is thus a reasonable hypothesis to consider that the ancestral eukaryotic MPP already had this property, which would have been crucial for adaptation of the ancestral endosymbiont. A first copy of OOP would also have been acquired from proteobacteria, at the time of the mitochondrial endosymbiosis, with a possible role of AMP-resistant α and γ-proteobacteria. A second acquisition of OOP probably occurred at the time of chloroplast endosymbiosis, with a possible role of a pathogenic Chlamydia, in agreement with the “Ménage à trois” hypothesis (MATH), in which pathogenic Chlamydiales provided critical help for maintenance of the chloroplast ancestor into the protist host (Greiner and Bock 2013; Ball et al. 2016; Cenci et al. 2016, 2017). As for OOP, PreP would have been acquired twice most probably from a resistant Terrabacteria or a Proteobacteria. The first copy would have been acquired at the time of mitochondrial endosymbiosis and the second copy at the time of the chloroplast endosymbiosis, with the first acquired copy being lost after the chloroplast endosymbiosis in photosynthetic eukaryotes. If this hypothesis is true, then the second acquired copy works not only in the chloroplast, but also in the mitochondria, as in *A. thaliana* where it has been experimentally demonstrated (Kmiec et al. 2014). The chloroplast counterpart of MPP, SPP would have been acquired during chloroplast endosymbiosis from an FCB bacteria with no evidence of contribution from resistant-bacteria.

Our results are based on a minimal group of bacteria for which experimental evidence of resistance to AMPs has been described, with a considerable and expected bias towards Enterobacteria. In particular, to our knowledge there are no reports of studies regarding possible resistance to AMPs among cyanobacteria, from which originated the chloroplast. On the other hand, we considered a whole bacterial genus as potentially antimicrobial resistant but functional evidence is not available for every single species, leading to possible over-interpretation that should however be minimal if orthologous conservation is a proxy for functional conservation.

In conclusion, AMP-resistant bacteria have OOP, MPP, and PreP homologs in the closest vicinity to their eukaryotic counterparts, which is consistent with their possible implication in the early stages of mitochondrial endosymbiosis and for OOP and PreP, also in the early stage of the chloroplast endosymbiosis. It is of note that very few SPP homologs from resistant-bacteria were found close to SPP in photosynthetic eukaryotes.

As Microsporidia lost their mitochondria after the divergence of Fungi, the absence of MPP and PreP, found exclusively in organelles is expected. As many OOP paralogs remain cytosolic, the fact that Microsporidia also lack OOP is consistent with a possible action of OOP on proteins that failed to be imported or that were released from mitochondria. The fact that the four other eukaryotes devoid of the four peptidases are all pathogens could suggest an unusual mitochondrial metabolism in those species.

With our previous finding that a certain class of HA-RAMPs and organelle TPs are likely evolutionary related, based on their shared physico-chemical properties, and their ability to functionally complement each other (Garrido et al. 2020; Caspari et al. 2021) the present phylogeny of organellar peptidases is fairly consistent with the hypothesis that the organelle-targeting machineries derive from an antimicrobial resistance (Wollman 2016; Caspari and Lafontaine 2021), through an import and destroy mechanism that has been documented in several studies. In that respect, the fact that the co-occurrence of MPP and PreP/OOP is higher in AMP-resistant α-proteobacteria/Terrabacteria compared to sensitive α-proteobacteria/Terrabacteria suggests that MPP and PreP/OOP were likely to be involved in the destroy steps. Moreover, we note that the Gram-negative γ-proteobacteria Pseudomonas and Acinetobacter are opportunistic pathogen that possess MPP, OOP, and SPP homologs closely related to their eukaryotic counterparts, making them model organism of choice that possess the major part of the proteolytic pathways for the degradation of AMP and AMP-derived TPs. In *Pseudomonas aeruginosa* HA-RAMP activity requires its binding to outer membrane proteins. This could probably promote HA-RAMP uptake and cytoplasma protrusion as it has been proposed for the HA-RAMP hRNase 7 with the outermembrane protein OprI (Lin et al. 2010), and for the HA-RAMP LL-37 with the porin OmpA (Lin et al. 2015). Other HA-RAMPs are actively internalized via import pump, like the bacteriocin pyocin S2 that can hijack the iron transporter FpvAI, a β-barrel TonB-dependent transporter (TBDTs) in *P. aeruginosa* (White et al. 2017). Such hijacking of TBDTs by bacteriocins is also observed in the Gram-negative phytopathogens *Pectobacterium* species in which pectocin M1 and M2 containing a ferredoxin domain can enter the bacterial cell through iron-uptake (Grinter et al. 2012, 2014). In those phytopathogens, FusC is associated with the TBDTs to promote the import of ferredoxin across the outer membrane (Grinter et al. 2018). This protease-associated import system capable of HA-RAMP uptake is widespread in Gram-negative bacteria (Grinter et al. 2019) and suggests that such association could have played a role in the establishment of an early protein import system upon endosymbiosis.

Further investigations are needed to estimate the evolutionary relationships between bacterial peptide uptake mechanisms in resistant bacteria and the TIM/TOM and TIC/TOC complexes. Indeed, several subunits of the extant translocons are of bacterial origin. For example, the protein core of TOC/TIC, composed of Toc75, Tic136, and Tic110, is thought to be derived from the bacterial β-barrel protein export system BAM/TAM, composed of BamA/TamA (Omp85 and Toc75 homologs) and TamB (Tic136 homolog) (Day and Theg 2018; Richardson and Schnell 2020). Similarly, mitochondrial outer membrane β-barrels (VDAC, TOM40, but also lineage-specific outer membrane β-barrels like ATOM in trypanosoma) are homologous and thought to derive from bacterial primitive channels (Pereira and Lupas 2018), and Tim23, Tim44 and Tim 14 of the TIM23 complex also have bacterial homologs (Rassow et al. 1999; Clements et al. 2009).

## Methods

### Peptidase Sequences and Proteomes

Reference sequences were selected for each of the four peptidases in the photosynthetic model plant *A. thaliana*: the two members of the PreP family PreP1, Prep2; the three MPP subunits: MPPα1, MPPα2, MPPβ; OOP and SPP, see supplementary table S1, Supplementary Material online for accession numbers. The search for homologs was performed against a database composed of the 8,340 nonvirus proteomes from the Uniprot reference database (released version 26 February 2020), including 291 archaea, 6,819 bacteria, and 1,230 eukaryote proteomes.

### Family Reconstruction

First, the proteome database was searched for significant hits (minimum query coverage of 70% and *E*-value <10^−5^) with BLASTP (standalone version 2.6.0) (Altschul et al. 1997) for each of the reference proteases (table 1). In order to retrieve remote homologs from the *A. thaliana* sequences, we built some HMM profiles for each peptidase with hmmbuild. Hits for the highly similar paralogs PreP1 and PreP2, MPPα1 and α2, MPPβ1 and β2 were merged. Hits were then clustered with the ETE3 toolkit (3.1.1), according to the major phylum of Archaea, Bacteria, and Eukaryota of the NCBI taxonomy. Clusters with <5 hits were merged with the clusters from sister phyla. The clusters used are listed in supplementary table S1, Supplementary Material online. Each cluster was then multiply aligned with MUSCLE v3.8.31 and the resulting multiple alignment was used to build the HMM profile with hmmbuild from hmmer-3.2.1 (hmmer.org). Obtained profiles are used to scan the proteome database. Hits with an *E*-value <10^−2^ covering >70% of at least one of the profiles were selected. Next, PFAM domains of interest were searched in the candidate sequences with hmmsearch (hmmer version 3.2.1), hits with an *E*-value <10^−2^ were selected. Candidates containing all the PFAM motifs present in the *A. thaliana* peptidase were considered as homologs. The presence of additional conserved domains was determined with hmmsearch (hmmer version 3.2.1) (*E*-value <10^−5^) on the entire PFAM database (3.1b2) (El-Gebali et al. 2019).

The M16 peptidase families was visualized by CLAN (Frickey and Lupas 2004) with the log(*E*-value) of the “all against all” BLAST between each pair of sequences.

### Motif Analysis

Logos of the multiple sequence alignments of the M16 and M3 motifs were generated with WebLogo 3.7.4 (Crooks et al. 2004).

### List of AMP-Resistant Bacteria

We performed a bibliographic screening for papers describing bacteria with a documented resistance to AMP. The genus from the identified bacteria is considered as an AMP-resistant bacterial genus. The list of the retrieved genus is given in supplementary data S1, Supplementary Material online, with corresponding references.

### Sample Set

To reduce the size of our dataset, a sample of homologs was generated by randomly picking one organism in each of the considered taxonomical group indicated in figure 2. All the peptidases from selected species are included in the sample set. One species per group of AMP-resistant bacteria was chosen. All homologs from *Chlamydomonas reihardtii* and *A. thaliana* were added.

### Species Tree

The phylogenetic relationships between the organisms included in our analysis were taken from the literature. The phylogeny of the Archaea as well as the placement of eukaryotes is based on Spang et al. (2017). The Eukaryota phylogeny is taken from Burki et al. (2020) and Torruella et al. (2015) for Opistokontha. The bacterial phylogeny is extracted from Castelle and Banfield (2018) and Zhu et al. (2019).

### Peptidase Tree

The concatenation of the aligned PFAM motifs identified in each peptidase family was performed with MAFFT v7.450 (Katoh and Standley 2013). Trees were reconstructed with the Approximately Maximum-Likelihood method FastTree (Price et al. 2010) (v2.1.11) with the JTT + CAT model. The local support values given by FastTree are based on the Shimodaira–Hasegawa (SH) test (Price et al. 2010).

We used the NCBI taxonomy retrieved with ETE3 toolkit to perform trees annotation. Tree display was performed with iTOL website (Letunic and Bork 2019). The phylogenetic analysis of the M16 family was performed with the sample set, using only the two first motifs M16 and M16C shared by the three M16 peptidases. For each family, MPP, SPP, PreP, and OOP, the alignments were performed with only the conserved motifs given in table 1. The sequences used as outgroups for the M16 peptidases are given in supplementary data S1, Supplementary Material online. The OOP tree was rooted with the midpoint method.

### Minimal Distance to Eukaryote

The evolutionary distance is estimated by the sum of the branch length between a given bacteria and its closest eukaryotic homolog. The topological distance corresponds to the number of internal nodes separating a bacterium to its closest eukaryotic homolog. Both distances were computed with python ETE3 toolkit (3.1.1). The pairwise distance corresponds to the log(*E*-value) Blastp between a bacterial sequence and its eukaryote best hit, with the BLAST version 2.6.0 (Altschul et al. 1997).

## Supplementary Material

Supplementary data are available at *Genome Biology and Evolution* online.

## Supplementary Material

evac101_Supplementary_Data

## Data Availability

Supplementary data S2, phylogenetic trees in newick format, all Python and R in-house scripts are available at https://github.com/UMR7141/Peptidase_Analysis.
