# Supplementary material for: The Evolutionary History of Peptidases Involved in the Processing of Organelle-Targeting Peptides
Source: Genome Biol Evol. 2022 Jun 27;14(7):evac101. doi: 10.1093/gbe/evac101 (PMC9291397; doi:10.1093/gbe/evac101)
Supplement: evac101_Supplementary_Data [file evac101_supplementary_data.zip › GBE-211218-SuppTables_Figures.R1.docx]

`

|  |  | MPPα | MPPβ | PreP | SPP | OOP |
| --- | --- | --- | --- | --- | --- | --- |
|  |  | NP_175610 | NP_186858 | Q9LJL3 | Q9FIH8 | Q94AM1 |
|  |  | NP_566548 | NP_850500 | Q8VY06 |  |  |
|  | HMM |  |  |  |  |  |
| Eukaryotes *(total hits)* |  | 518 | 509 | 162 | 172 | 211 |
|  | Viridiplantae | 213 | 304 | 149 | 150 | 178 |
|  | Sar | - | 34 | - | - | - |
|  | Metazoa | 218 | 110 | - | - | - |
|  | Fungi | 62 | 53 | - | - | - |
|  | other eukaryotes | 25 | - | 13 | 22 | 33 |
| Bacteria *(total hits)* |  |  | 1 | 345 | 109 | 281 |
|  | Terrabacteria | - | - | 267 | - | 118 |
|  | Proteobacteria | - | - | 61 | 49 | 146 |
|  | FCB | - | - | - | 51 | - |
|  | other bacteria | - | - | 17 | 9 | 17 |
|  |  |  |  |  |  |  |
| Archaea *(total hits)* |  | 0 | 0 | 0 | 0 | 0 |
|  | Others | - | 9 | - | - | - |

**Supplementary Table 1:** For each peptidase, the composition of the different taxonomical HMM are given. The number of significant BLAST hits for each *A. thaliana* peptidase is given for each HMM, a hyphen indicates that no HMM was built for that taxon (see Methods for the selection criteria). For each domain, the total number of hits is given. The HMM “Others” is composed of 1 bacterial sequence and 8 eukaryotic sequences. The Uniprot ID of each sequence used for the first BLAST search is given below the peptidase name. There are two paralogs for MPP subunits a and b, and for PreP.

|  | **MPP top200** | | | | **PreP top200** | | | | **SPP top200** | | | | **OOP top200** | | | |
| --- | --- | --- | --- | --- | --- | --- | --- | --- | --- | --- | --- | --- | --- | --- | --- | --- |
|  | *B* | *T* | *S* | *u* | *B* | *T* | *S* | *u* | *B* | *T* | *S* | *u* | *B* | *T* | *S* | *u* |
| Archaea | 1 | 4 | 1 | 5 | 0 | 0 | 0 | 0 | 0 | 0 | 0 | 0 | 1 | 6 | 0 | 6 |
| Terrabacteria | 21 (10) | 52 | 0 | 70 (10) | 61 (18) | 35 (4) | 69 | 134 (45) | 1 | 1 | 0 | 2 | 7 | 0 | 0 | 7 |
| Cyanobacteria | 85 | 4 | 0 | 86 | 3 | 0 | 0 | 3 | 2 | 0 | 0 | 2 | 106 | 12 | 119 | 120 |
| Proteobacteria | 39 (1) | 84 (46) | 38 | 161 (47) | 103 | 92 (2) | 69 | 133 (1) | 1 | 80 (8) | 24 | 104 (8) | 79 (27) | 97 (11) | 39 (1) | 167 (38) |
| *a-proteobacteria* | 12 | 12 (2) | 115 (55) | 139 (57) | 19 | 20 | 19 | 21 | 4 | 0 | 0 | 4 | 0 | 6 | 25 (1) | 31 (1) |
| PVC (*Chlamydiae*) | 6 | 26 | 0 | 31 | 5 (3) | 12 (10) | 1 | 12 (10) | 22 | 1 | 0 | 22 | 13 | 40 (1) | 16 (1) | 44 (1) |
| FCB | 13 | 17 | 0 | 23 | 1 | 4 | 39 | 4 | 197 | 75 | 167 | 311 | 27 | 0 | 0 | 27 |
| Other bacteria | 34 | 36 | 0 | 63 | 11 | 34 (1) | 3 | 34 (1) | 3 (1) | 15 | 7 | 21 (1) | 1 | 5 | 1 | 6 |

**Supplementary Table 2**. Composition of the set of homologs in prokaryotes, the most closely related to eukaryotic peptidases MPP, PreP, SPP and OOP, named top200 in the main text. For each peptidase, the number of homologs per group is given according to three metrics: the evolutionary distance (B), the topological distance (T) and the pairwise similarity distance (S). The union of the three metrics is also given (u), named pan-top200 in the main text. The number of AMP-resistant bacteria in each group is given in parenthesis.


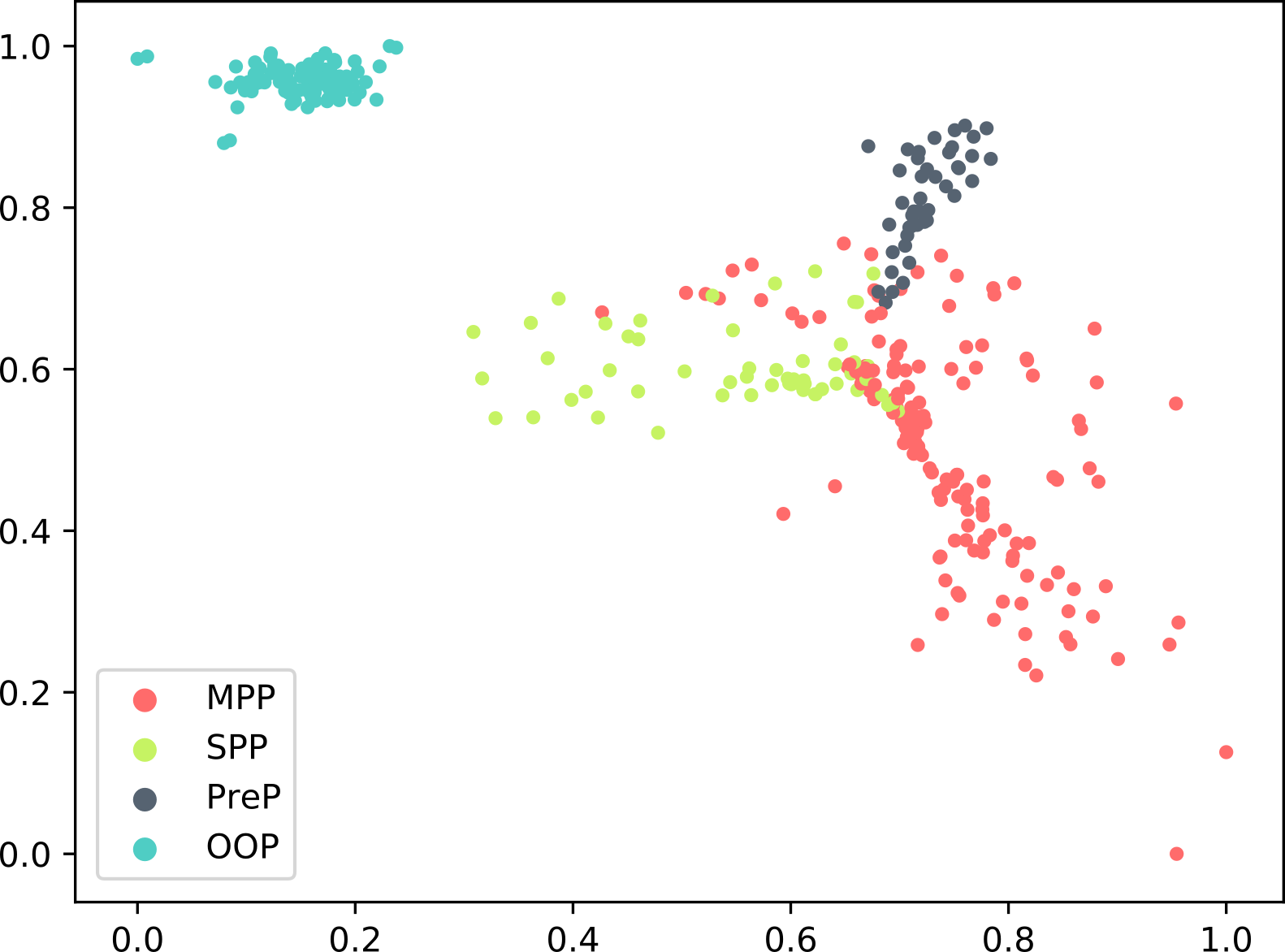


**Supplementary Figure 1.** CLAN analysis of the four peptidases shows family M16 with MPP (in red), SPP (in green) and PreP (in grey), distinct from the OOP from the M3 family (in blue)


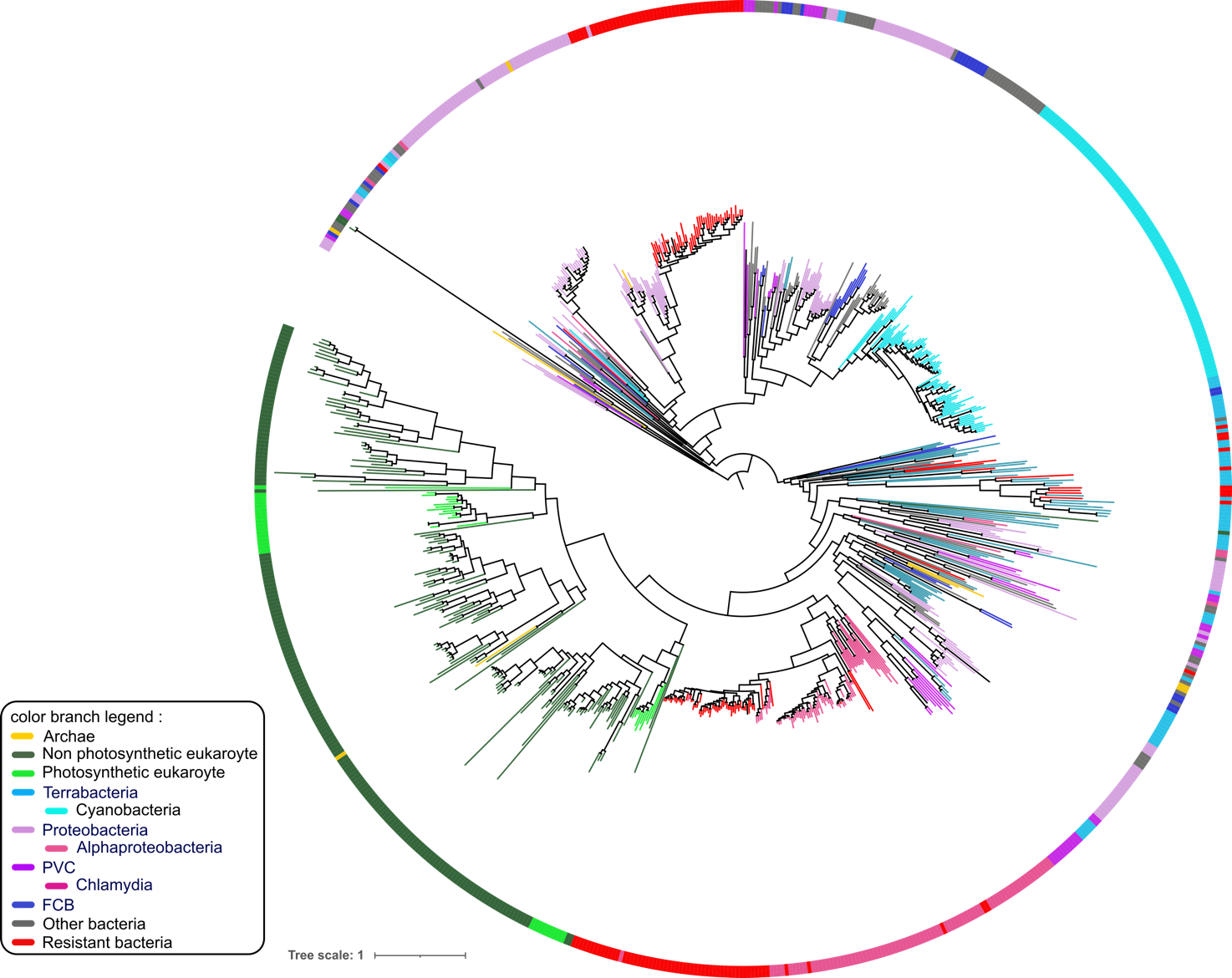


**Supplementary Figure 2:** Tree of 200 eucaryote MPP peptidases chosen randomly, but making sure to take sequences from each taxonomic group and 578 prokaryote homologs identified as the closest to eukaryotes (see Methods). The eucaryotic MPP are a sister group of α-proteobacterial MPP homologs, including peptidases from AMP-resistant α-proteobacteria.

##
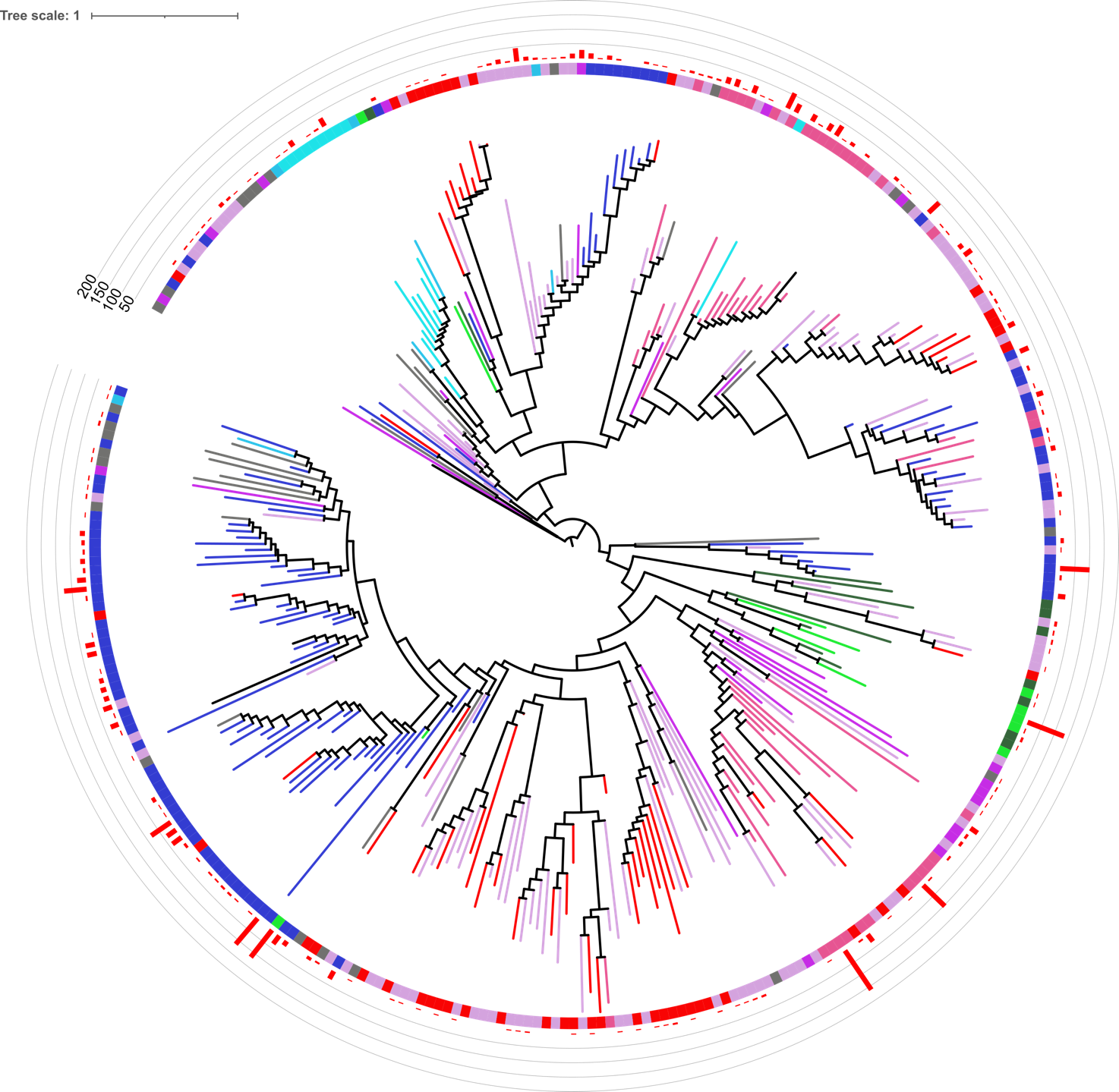


## **Supplementary Figure 3A.** SPP tree where the nodes whose leaves belongs to the same NCBI taxonomical group are collapsed. Same legend as Figure 3.


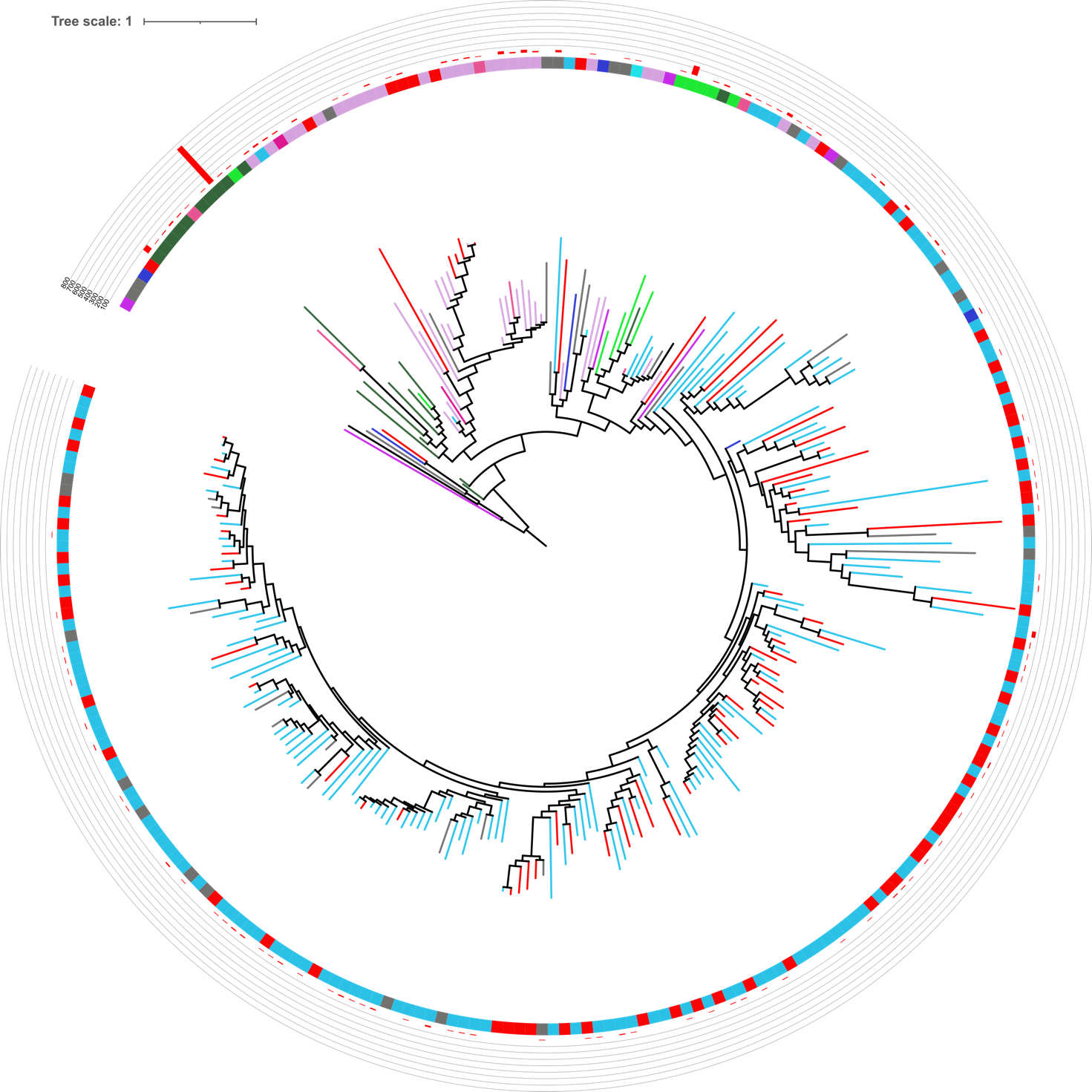


## **Supplementary Figure 3B.** PreP tree where the nodes whose leaves belongs to the same NCBI taxonomical group are collapsed. Same legend as Figure 3.


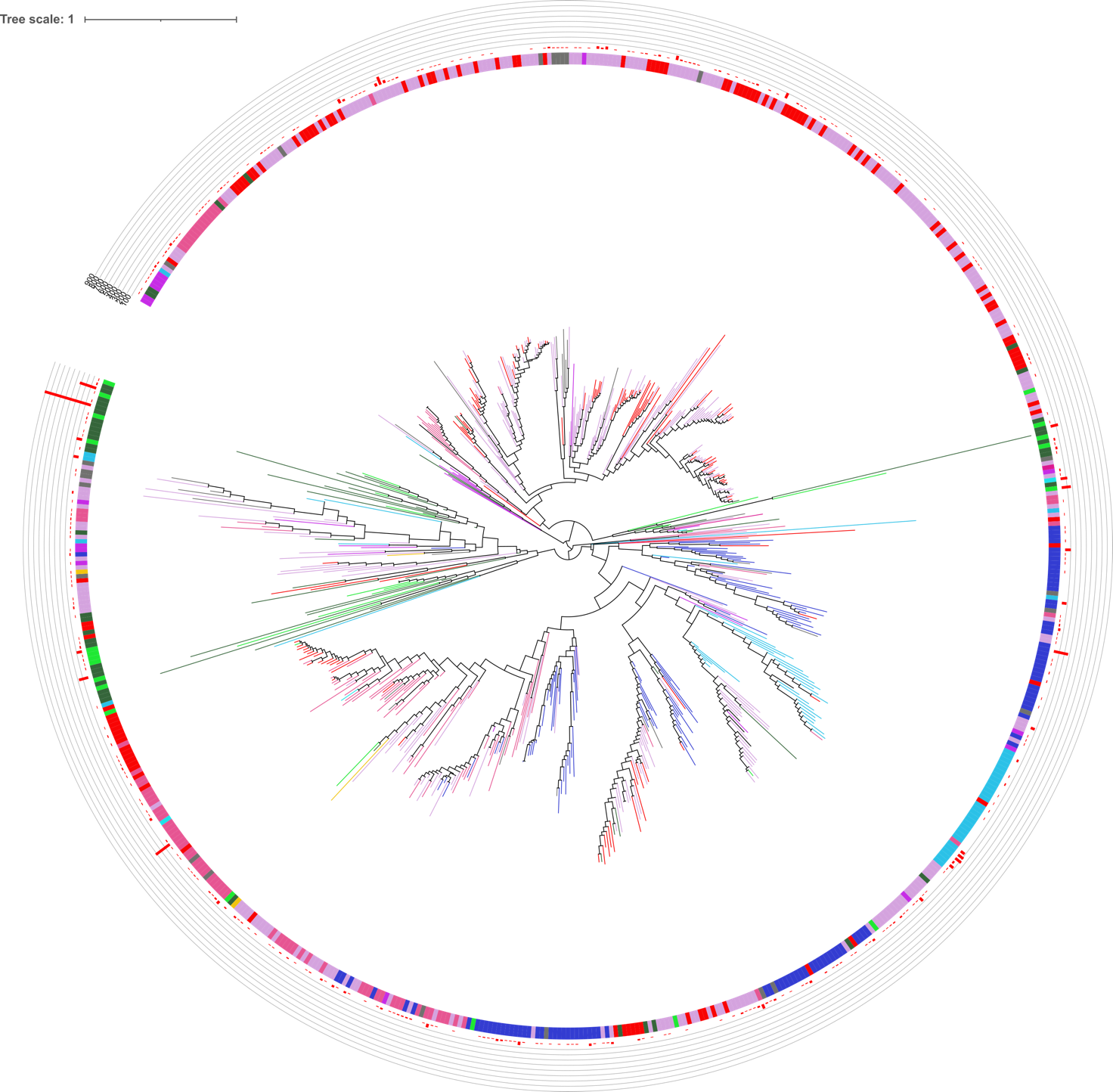


## **Supplementary Figure 3C.** OOP tree where the nodes whose leaves belongs to the same NCBI taxonomical group are collapsed. Same legend as Figure 3.


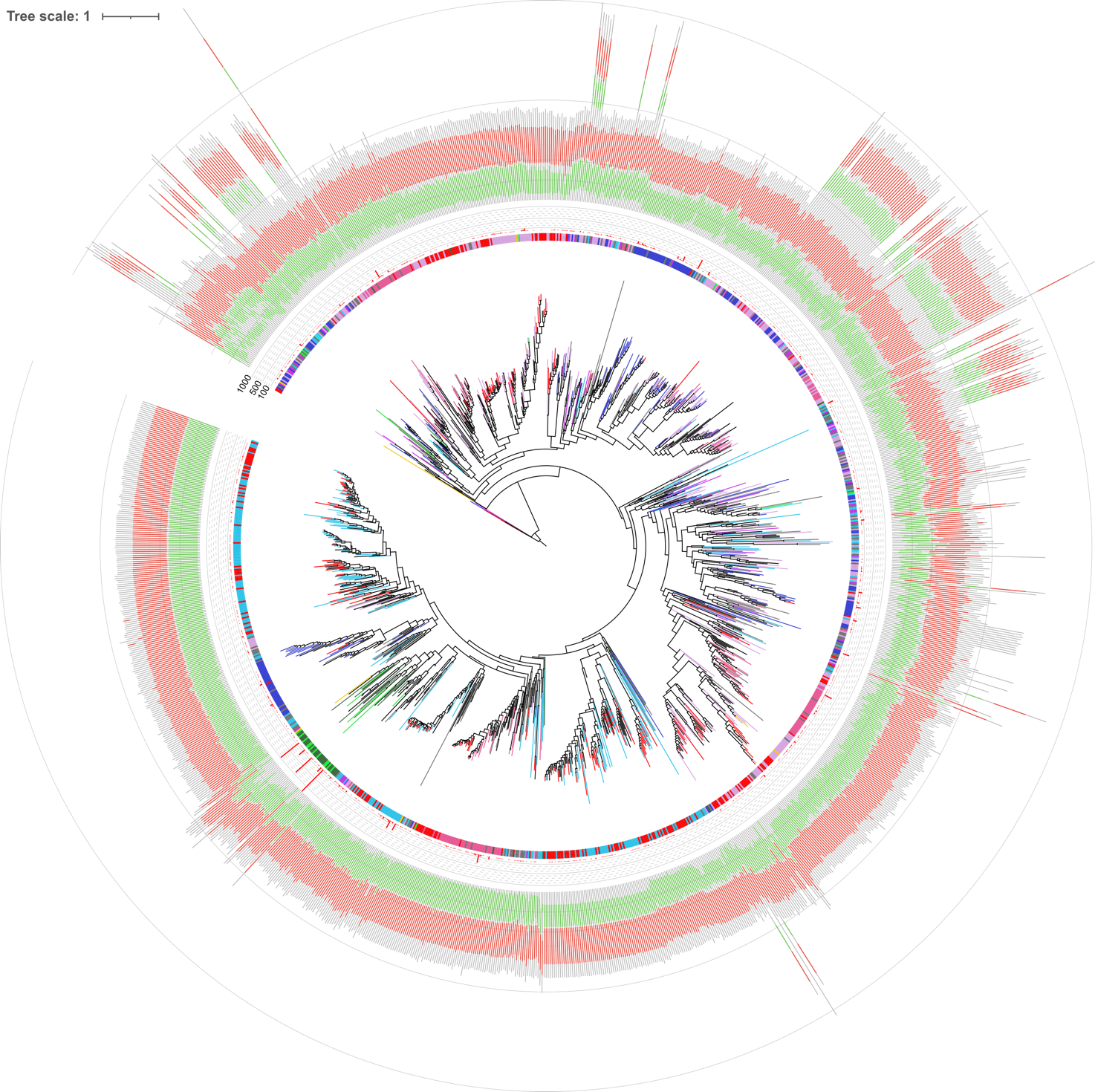


**Supplementary Figure 4A:** Same MPP tree as presented in Figure 3 with a schematic representation of PFAM motifs M16 and M16C in green and red respectively, for each sequence in the tree. The length of the sequence is represented by a grey line.


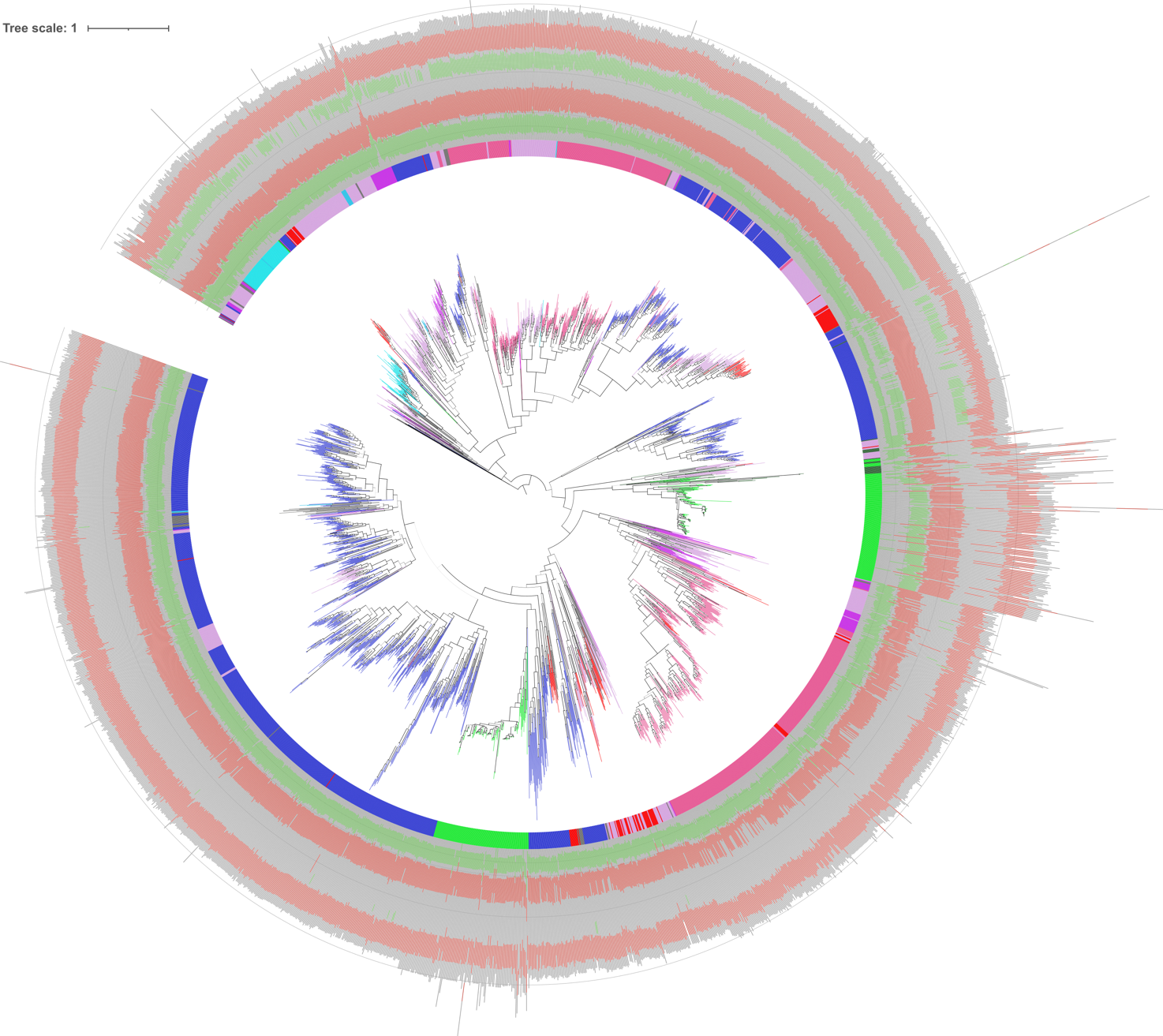


**Supplementary Figure 4B:** Same SPP tree as presented in Figure 5 with a schematic representation of PFAM motifs M16 in green and M16C in red for each sequence in the tree. Note that the tree was built on the multiple alignment of the first M16 domain and the 2 M16C domain only. The length of the sequence is represented by a grey line.


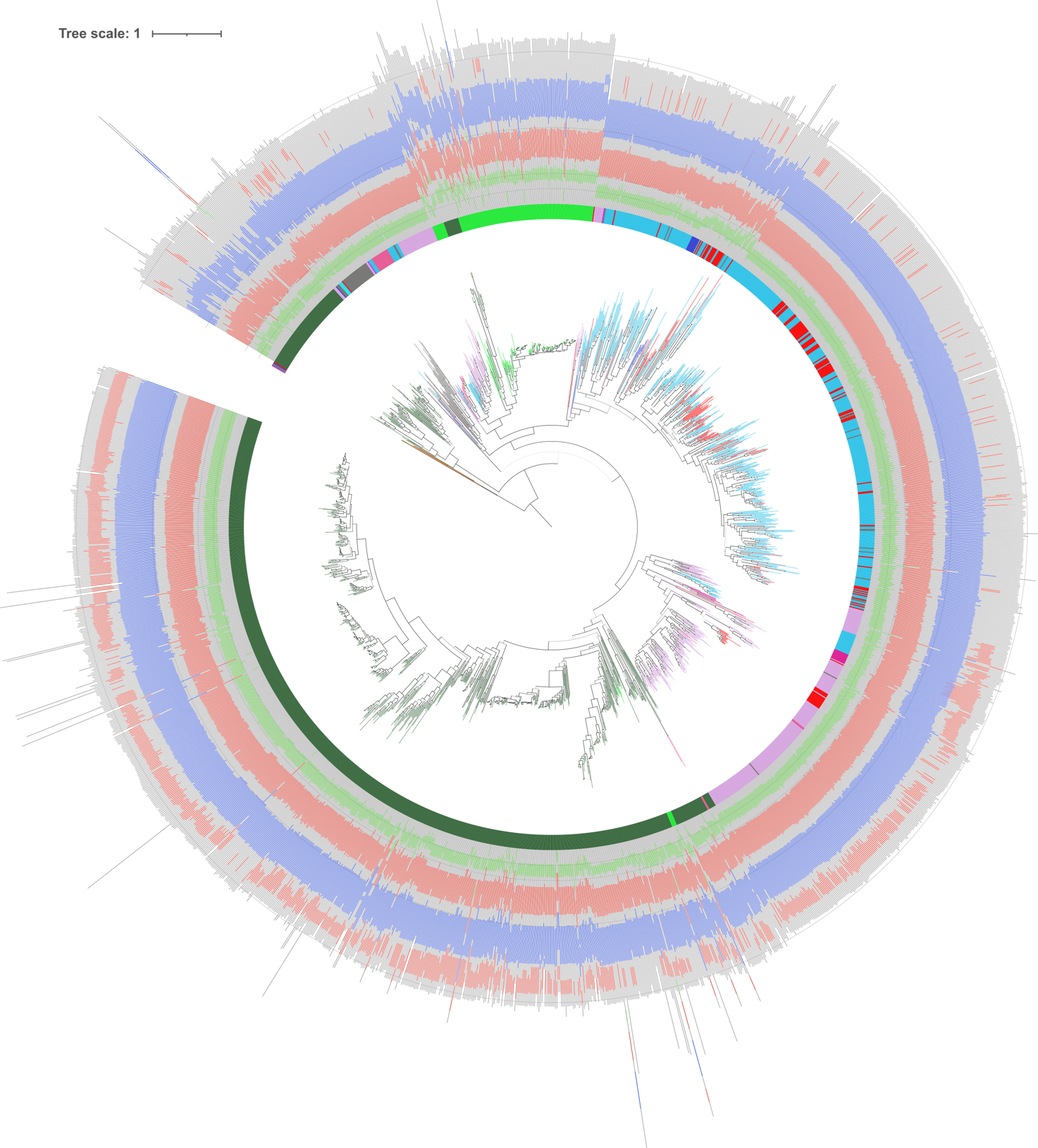


**Supplementary Figure 4C:** Same PreP tree as presented in Figure 4 with a schematic representation of PFAM motifs M16 in green, M16C in red and M16C associated in blue for each sequence in the tree. Note that the tree was built on the multiple alignment of the three first domains only. PreP homologs in the NPSeuka subtree and in its sister bacterial group comprises 4 domains (ordered as M16/M16C/M16C-associated/M16C) whereas the PreP homologs in the PSeuka subtree and in its sister bacterial group, as well as the homologs in the Baseuka subtree comprises only 3 domains (ordered as M16/M16C/M16C-associated). Based on this character, the Baseuka subtree could correspond either to highly diverged homologs from the PSeuka subtree (same domain architecture) or to highly diverged paralogs from the NPSeuka subtree, in which the 4^th^ M16C domain would have diverged beyond recognition. The length of the sequence is represented by a grey line.


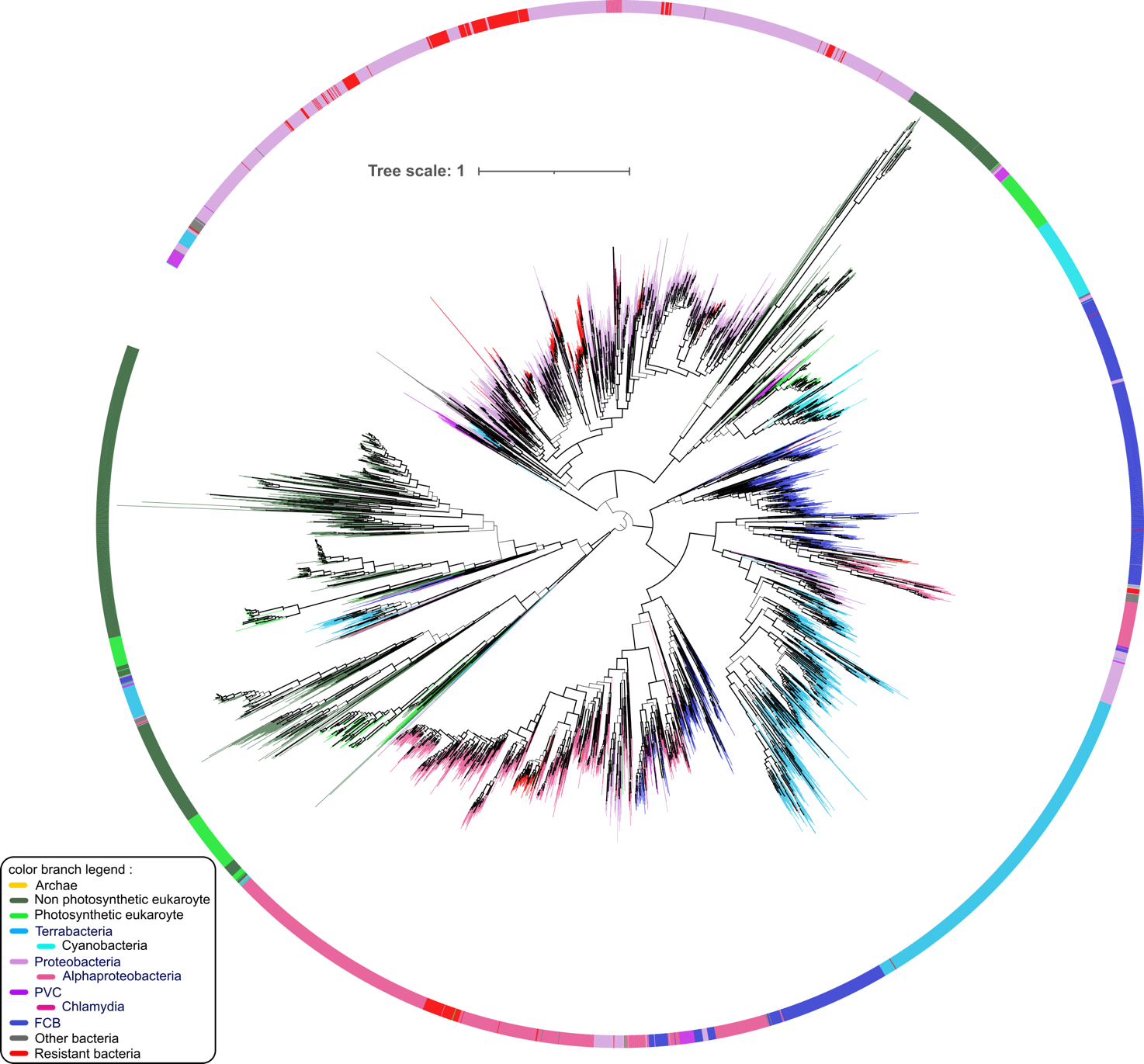


**Supplementary Figure 5:** OOP phylogenetic tree with only the 1071 eukaryotic homologs predicted to be addressed to mitochondria and/or chloroplast. If bacterial paralogs exist, the one closest to an eukaryotic homolog was chosen, according to the pairwise distance (see Methods).


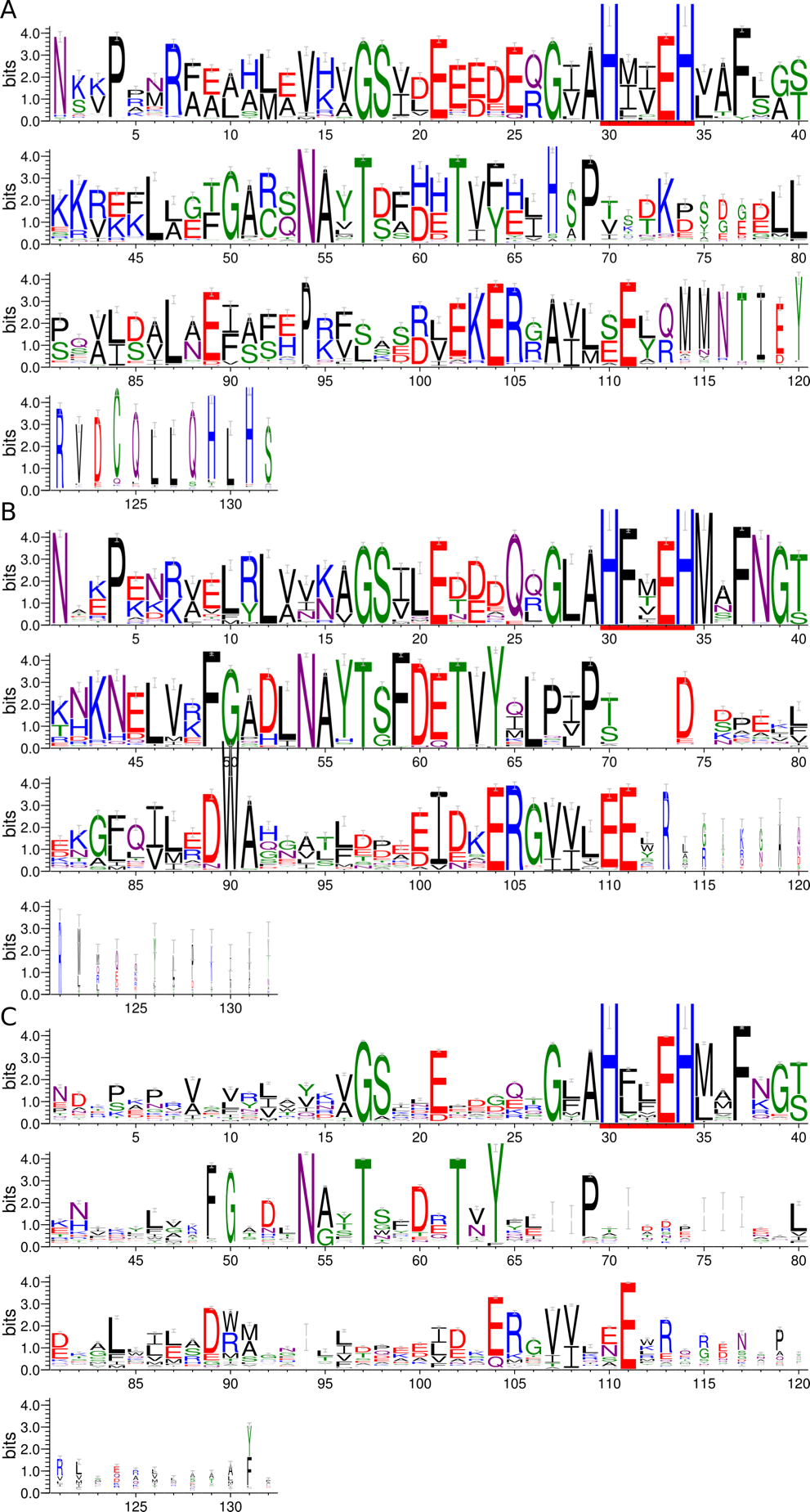


## **Supplementary Figure 6**

## Sequence logos of the M16 Motif in SPP homologs. A) Homologs in eukaryotes. B) The closest to eukaryotes 200 bacterial homologs according the similarity distance C) All homologs in bacteria. The first position corresponds to the first residue of the M16 motif of *A. thaliana* SPP. The overall height of the stack at each position reflects conservation, the height of residues reflects their relative frequency and the width of the stack is inversely proportional to the number of gaps at that position. Residues are colored according to their chemical properties, green: polar, purple: neutral, basic: blue, acidic: red, hydrophobic: black. The HXXEH motif of the catalytic site is underlined in red.


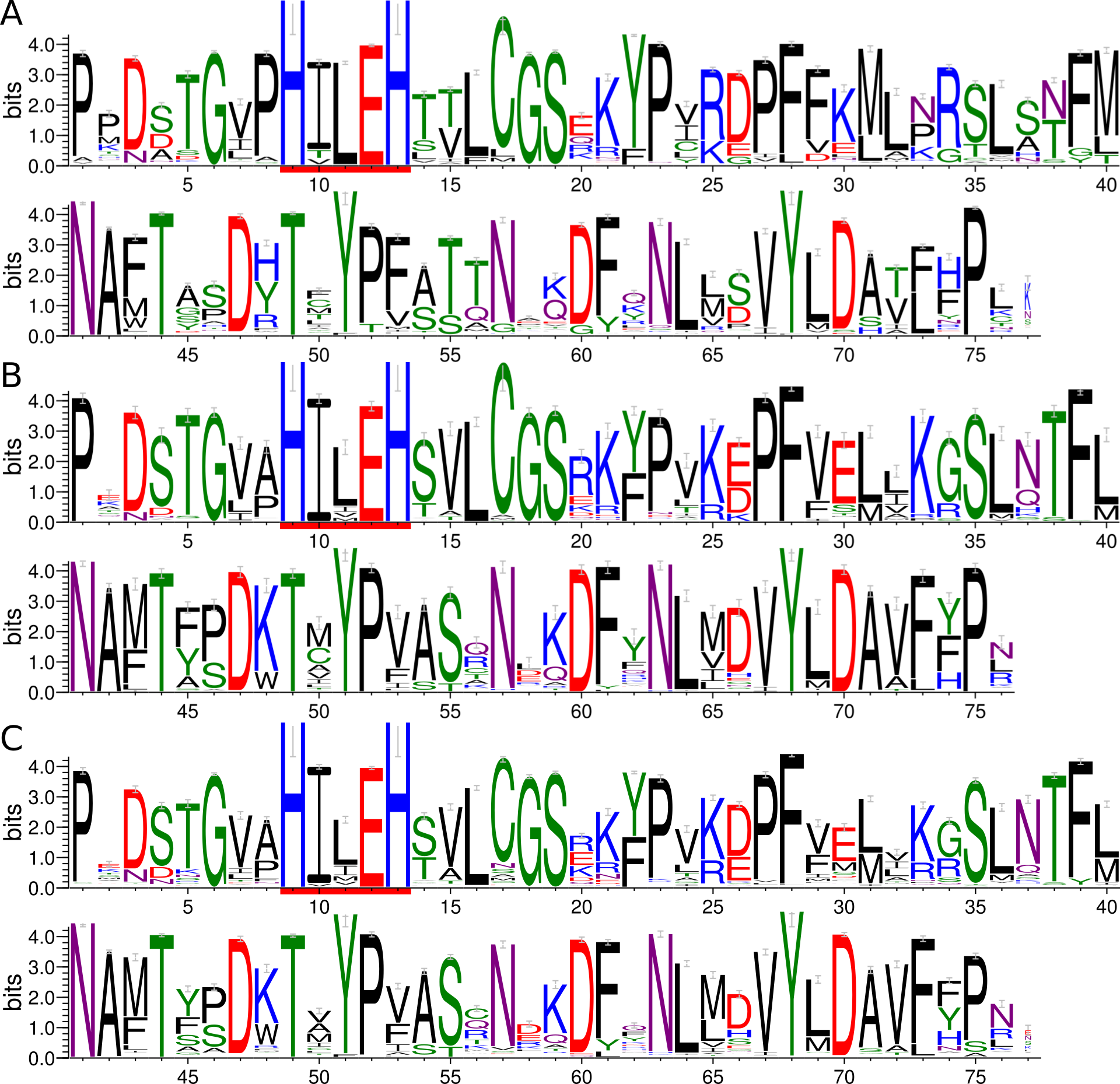


## **Supplementary Figure 7**

## Sequence logos of the M16 Motif in PreP homologs. The first position corresponds to the first residue of the M16 motif of *A. thaliana* PreP2. Same legend as Supplementary Figure 6.


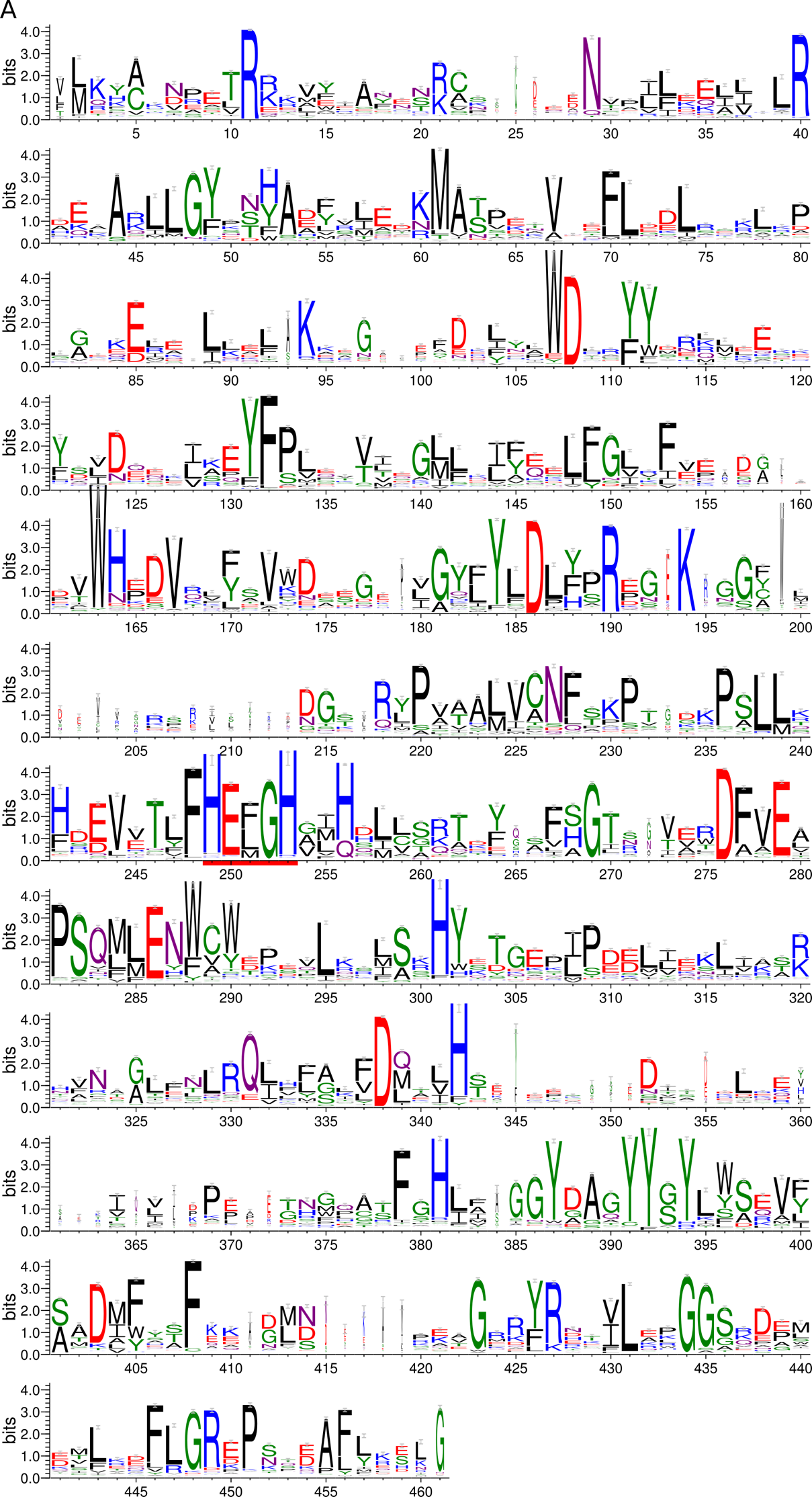


## **
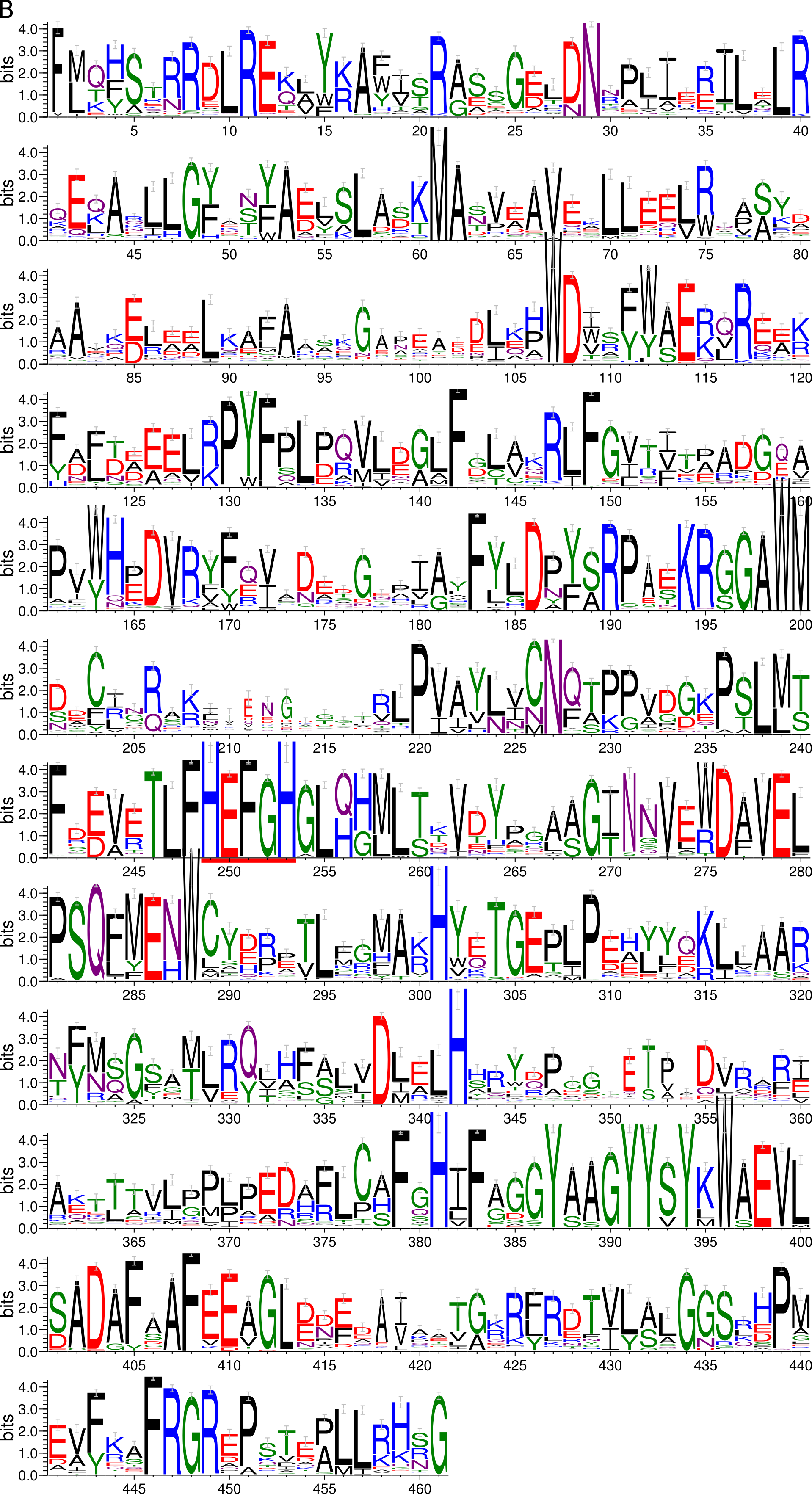
**

## **
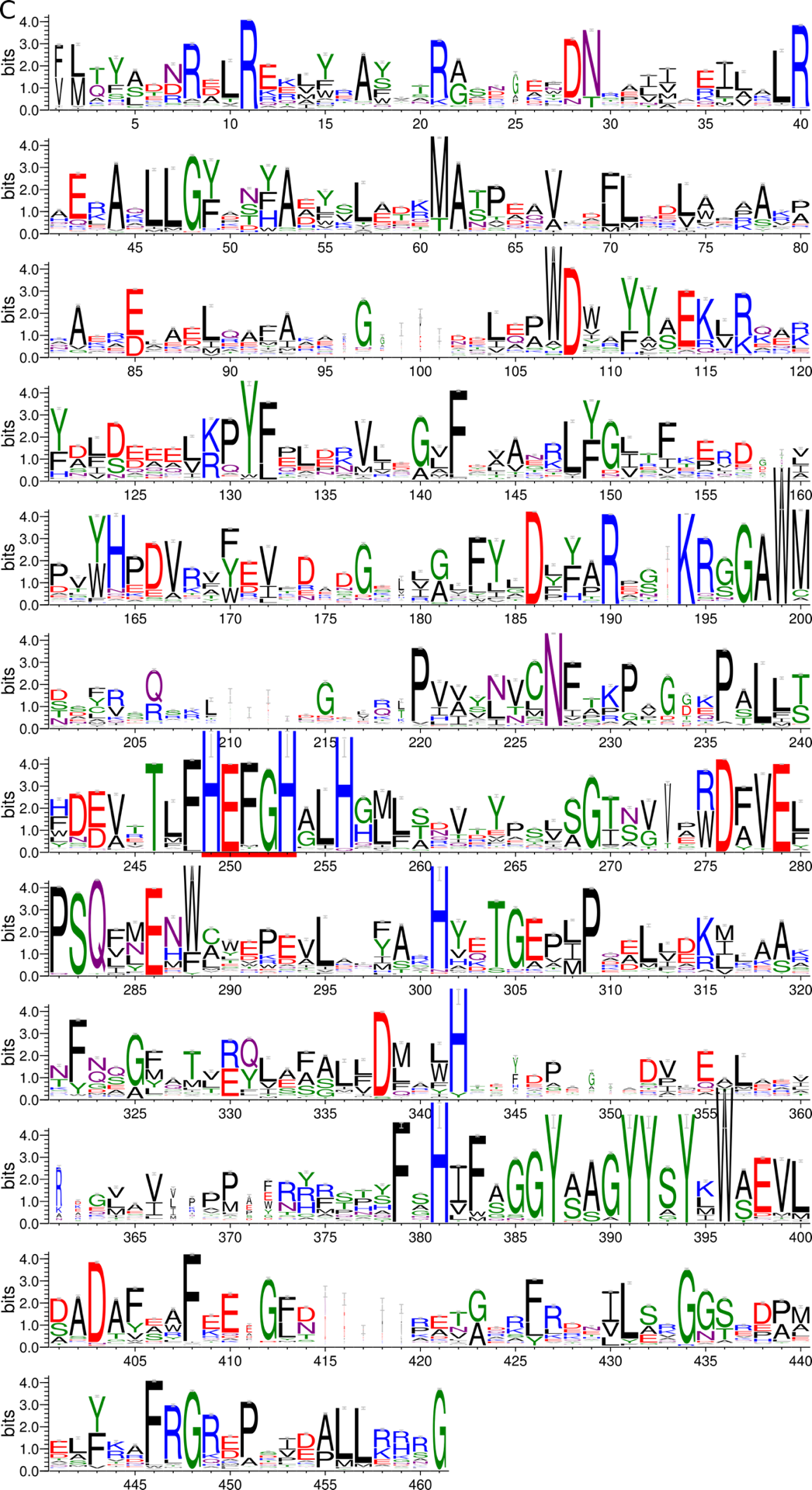
**

## **Supplementary Figure 8** Sequence logos of the M16 Motif in OOP homologs. The first position corresponds to the first residue of the M16 motif of *A. thaliana* OOP. Same legend as Supplementary Figure 6.


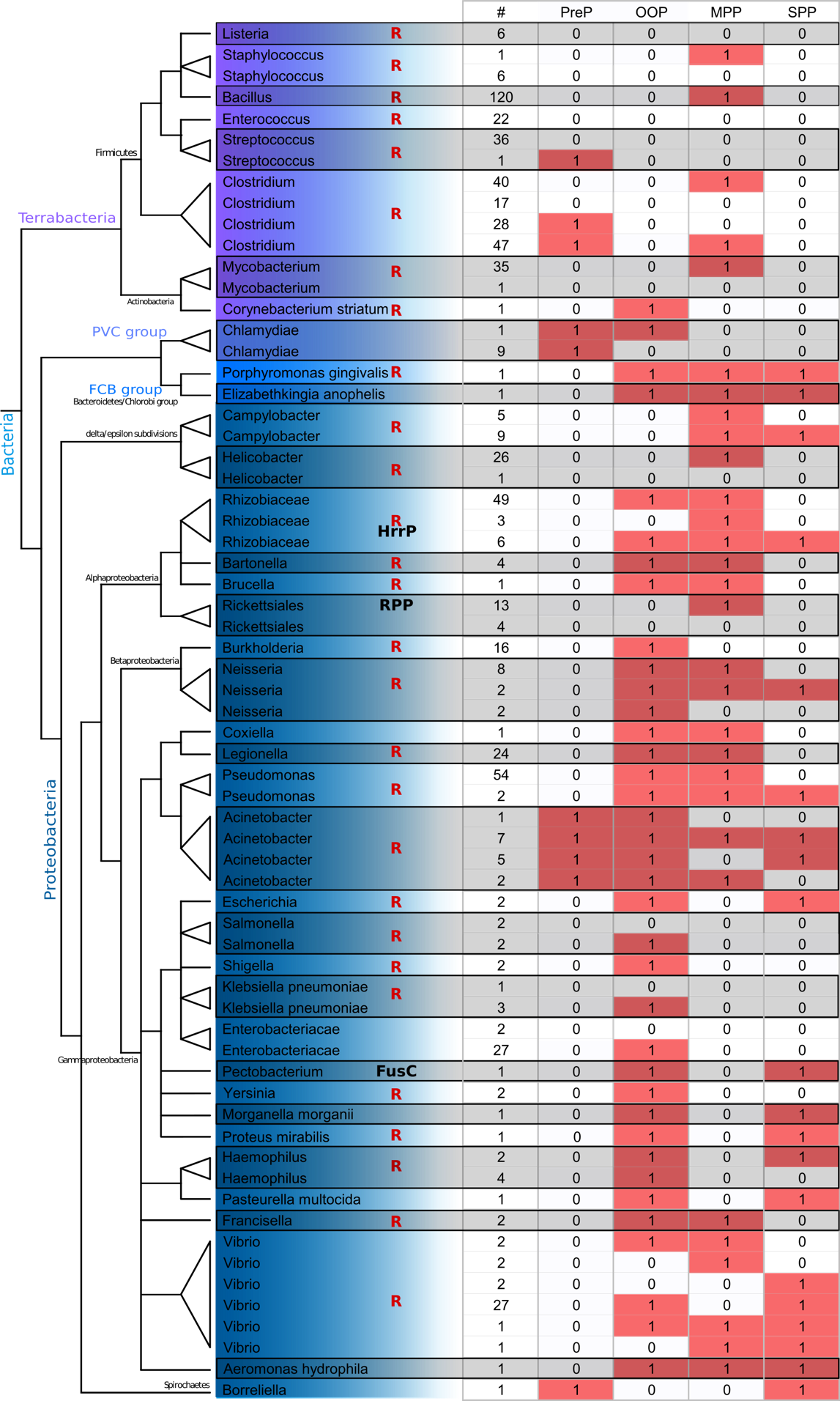


## **Supplementary Figure 9**

Taxonomic distribution of the four peptidases among AMP-resistant bacteria documented in this study, indicated by a red R, as well as Rickettsiales species for their proximity to mitochondria and Chlamydiales for the proposed role during the chloroplast endosymbiosis (see text). The cladogram represents the phylogenetic relationships between the different taxonomic groups (see the Phylogeny Section in Methods for the reference trees used). Each line indicates a distinct peptidase profile of presence (1) or absence (0) of the four peptidases. The first column indicates the number of species presenting the presence/absence profile. Column 2 to 5 indicates the presence/absence of MPP, PreP, SPP and OOP respectively. PVC: superphylum composed of *Planctomycetes*, *Verrucomicrobia* and *Chlamydiae.* FCB: superphylum composed of *Fibrobacteres, Chlorobi and Bacteroidetes*.
